# The relevance of Brownian relaxation as power absorption mechanism in Magnetic Hyperthermia

**DOI:** 10.1038/s41598-019-40341-y

**Published:** 2019-03-08

**Authors:** Teobaldo E. Torres, Enio Lima, M. Pilar Calatayud, Beatriz Sanz, Alfonso Ibarra, Rodrigo Fernández-Pacheco, Alvaro Mayoral, Clara Marquina, M. Ricardo Ibarra, Gerardo F. Goya

**Affiliations:** 1 Instituto de Nanociencia de Aragón (INA), Universidad de Zaragoza, C/Mariano Esquillor s/n, CP 50018 Zaragoza, Spain; 2 Laboratorio de Microscopias Avanzadas (LMA), Universidad de Zaragoza, C/Mariano Esquillor s/n, CP 50018 Zaragoza, Spain; 3 Div. Resonancias Magnéticas, Centro Atómico de Bariloche/CONICET, S.C 8400 Bariloche, Argentina; 4 School of Physical Science and Technology, Shanghai Tech University. 393 Middle Huaxia Road, 201210 Pudong, Shanghai, China; 5 Departamento de Física de la Materia Condensada, Facultad de Ciencias, Universidad de Zaragoza, CP 50009 Zaragoza, Spain; 6 Instituto de Ciencias de Materiales de Aragón (ICMA), Consejo Superior de Investigaciones Científicas (CSIC) - Universidad de Zaragoza, Zaragoza, Spain

## Abstract

The Linear Response Theory (LRT) is a widely accepted framework to analyze the power absorption of magnetic nanoparticles for magnetic fluid hyperthermia. Its validity is restricted to low applied fields and/or to highly anisotropic magnetic nanoparticles. Here, we present a systematic experimental analysis and numerical calculations of the specific power absorption for highly anisotropic cobalt ferrite (CoFe_2_O_4_) magnetic nanoparticles with different average sizes and in different viscous media. The predominance of Brownian relaxation as the origin of the magnetic losses in these particles is established, and the changes of the Specific Power Absorption (SPA) with the viscosity of the carrier liquid are consistent with the LRT approximation. The impact of viscosity on SPA is relevant for the design of MNPs to heat the intracellular medium during *in vitro* and *in vivo* experiments. The combined numerical and experimental analyses presented here shed light on the underlying mechanisms that make highly anisotropic MNPs unsuitable for magnetic hyperthermia.

## Introduction

The specific power absorption (SPA), also known as specific absorption rate (SAR) or specific loss power (SLP), quantifies the power absorbed by a system of MNPs due to magnetic losses, taking place when an alternate magnetic field (AMF) it is applied to the sample. Magnetic losses are the main physical phenomena involved in magnetic hyperthermia treatments (MHT) to target and kill cancerous cells. The physics behind this mechanism of heating is related to the structural and magnetic parameters of the MNPs (namely the effective anisotropy constant K_eff_, saturation magnetization M_S_, average particle size 〈d〉) and to the viscosity of the medium (η). There are no simple analytical solutions for the SPA under general conditions. Many accepted models aim to calculate numerically the time-dependent magnetization as a function of the applied magnetic field, i.e., the hysteresis loop, as its area is the energy absorbed by the MNPs (i.e., the heat released to the environment) during a single AMF cycle. A study by J. Carrey *et al*.^1^ having the equilibrium functions, the Stoner-Wohlfarth model-based theories and the linear response theory (LRT)^2,3^ as starting point to describe the magnetic relaxation dynamics, has accounted for the power absorbed in the absence of Brownian relaxation. More realistic models developed by N.A. Usov *et al*.^4^ and H. Mamiya^5,6^ consider both Brownian and Neel relaxation to describe the magnetization dynamics of a single-domain nanoparticle by the stochastic Landau-Lifshitz equation.

Given the mathematical complexity of the stochastic approach, analytical expressions may be obtained only in some limits of the model. A frequently used approximation is to assume that the applied field H_0_, is small in comparison to the anisotropy field (H_K_) of the MNPs and therefore, the magnetic Zeeman contribution to the total energy of the system can be neglected. In this way, H_0_ does not distort the energy barrier that separates the two possible states between which the MNPs magnetic moment fluctuates (Néel relaxation). Within this assumption, the LRT makes use of the effective relaxation time that results when considering both Néel and Brown relaxations as independent mechanisms^2,3,7^. For MNPs with high effective anisotropy (i.e. H_k_ ≫ H_0_) the power absorption is driven by the rotation of the particles due to the magnetic torque at moderate or even high fields (which has been defined by N.A. Usov *et al*.^4^ as viscous mode). For MNPs with moderate anisotropies (e.g. iron oxides) the experimental values of H_0_ satisfying the LRT are roughly ${{\rm{H}}}_{0}\lesssim 10\,\mathrm{kA}/m$ ^4^.

With the aim of analyzing the validity of the LRT in highly anisotropic systems, the SPA of a series of cobalt-ferrite MNPs has been studied. Bulk cobalt ferrite has the highest magnetocrystalline anisotropy among all spinel ferrites, with a magnetocrystalline anisotropy constant K_1_ = 2 × 10^5^ J/m^3^. First, the SPA has been calculated, for MNPs of different average diameters assuming that their anisotropy and saturation magnetization have the values of the bulk CoFe_2_O_4_. With these input values, the simulations have been performed in a wide range of H_0_ and *f*, obtaining the dependencies of the SPA on these parameters. The SPA has also been measured in a series of Co-ferrite MNPs (with average diameters 〈d〉 between 5 and 25 nm) dispersed in hexane, in magnetic fields of amplitude up to 24 kA/m, and frequencies up to 828 kHz. The experimental SPA results have been compared with the numerical simulations carried out taking as input for our calculations the physicochemical parameters (size, magnetic anisotropy, and magnetization) obtained from the respective structural and magnetic characterization of the MNPs previously reported^8^. As far as we know, the good agreement observed between simulations and measurements constitutes the first experimental confirmation of the validity range of the LRT for highly anisotropic MNPs, establishing the strong correlation of the frequency and strength of the magnetic field in a given experiment, with the physicochemical parameters of a given MNPs suspension. Therefore, besides establishing the frequency and magnetic field strength for which the LRT is valid, our numerical simulations make it possible to find the optimal MNPs, with magnetic and structural parameters such that result in the maximum SPA for fixed experimental magnetic field amplitude (H_0_) and frequency (*f*) conditions.

When the magnetic anisotropy of MNPs is such that the energy barrier required to flip the magnetic moments is much larger than thermal energy at room temperature, the Brownian rotation is the predominant mechanism for magnetic relaxation^4^. In this situation, the hydrodynamic diameter of the MNPs and the viscosity of the medium are key parameters to determine the SPA. To explore the actual influence of these parameters on SPA in the case of Co-ferrite MNPs we performed systematic numerical simulations in media with different viscosities, finding good agreement with experimental SPA values measured. *In vitro* hyperthermia experiments were carried out on a culture of MNPs-loaded cells to assess the relevance of Brownian relaxation. Once inside the cell, MNP aggregates (whose presence was confirmed by Focused Ion Beam-FIB 3D reconstruction) are not free to rotate, due to the high viscosity of the medium. The lack of Brownian relaxation would explain the absence of heating observed in our experiments.

## Results and Discussion

### Numerical simulations of power absorption

We performed numerical simulations within the ‘classical’ SPA (CSPA) model, applied to magnetic colloids by Rosensweig^7^, which considers that both Néel and Brown relaxations are the main mechanisms for magnetic relaxation. As shown in the Supplementary Information, within the CSPA model for the magnetic relaxation of the MNPs the out-of-phase component *χ*″ of the magnetic susceptibility under an alternating magnetic field of amplitude H_0_ and frequency *f* is given by:

1 $$\chi ^{\prime\prime} ={\chi }_{0}\frac{2\pi f\tau }{[1+{(2\pi f\tau )}^{2}]}$$

where χ_0_ is the equilibrium susceptibility of the MNPs. The CSPA model assumes that the Néel and Brown relaxation are independent processes so that the effective relaxation time τ_*eff*_ of a single-domain MNP can be expressed as:

2 $${\tau }_{eff}^{-1}={\tau }_{N}^{-1}+{\tau }_{B}^{-1}$$

where

3 $${\tau }_{N}={\tau }_{0}{e}^{\frac{K{V}_{M}}{{k}_{B}T}}\,{\rm{and}}\,{\tau }_{B}=\frac{3\eta {V}_{h}}{{k}_{B}T}$$

are the Néel and Brown relaxation times and V_M_ and V_h_ are the magnetic and hydrodynamic volumes, respectively. The parameter *τ*_0_ can be considered as an ‘attempt time’ having values of ~10^−9^–10^−11^ s. General considerations about the origin of these two mechanisms show that they cannot be independent because the physical rotation (i.e. Brownian relaxation) of any particle respect to a fixed spatial coordinate system implies a change on the direction of the magnetic moment (i.e. Néel relaxation). For Co-ferrite nanoparticles, the large value of the magnetocrystalline anisotropy constant of this material gives, for particle sizes d > 5–6 nm, a large contribution from KV to the exponential in Eq. (3) that makes τ_N_ exceedingly large (see Fig. S3 in the Supplementary Information). Under these conditions, Brownian relaxation, is expected to dominate and τ_*eff*_ in Eq. (2) is given by a single contribution. For sizes d < 5–6 nm the two contributions to the relaxation must be considered.

Considering the above arguments and assuming a Gaussian size distribution for the MNPs, the CSPAM yields an expression for the power absorption of this ensemble of MNPs under an applied magnetic field of amplitude H_0_ and frequency *f* of the form (see the Supplementary Information).

4 $$SPA\,({\rm{d}})={\int }_{0}^{\infty }\,{\mu }_{0}f\pi {H}_{0}^{2}{\chi }_{0}\frac{2\pi f{\tau }_{eff}}{{(2\pi f{\tau }_{eff})}^{2}+1}\times \frac{1}{w\sqrt{\pi /2}}ex{p}^{[-2{(\frac{{\rm{d}}-\langle {{\rm{d}}}_{0}\rangle }{w})}^{2}]}\,d{\rm{d}}$$

where 〈d_0_〉 is the statistical mean value of the particle diameter, *w* gives the size distribution width (full-width at half maximum), *χ*_0_ is the susceptibility of an ensemble of particles in the equilibrium, and *χ*_0_ is the magnetic permeability on the free space.

To explore the validity of the model in purely Brownian systems, we have numerically calculated the SPA using Eq. (4). The simulations were performed for colloids of Co-ferrite MNPs of diameter 〈d〉 such as 1 ≤ 〈d〉 ≤ 100 nm and having a size distribution width *w* = 1.48 nm (see Supplementary Information). The carrier liquid was hexane (η = 2.94 × 10^−4^ kg/ms)^8^. The saturation magnetization and effective anisotropy constant were assumed to be those of the bulk CoFe_2_O_4_ phase (i.e., M_S_ = 4.2 × 10^5^ A/m, and K_eff_ = 2 × 10^5^ J/m^3^)^9^. From a previous physical characterization reported elsewhere^8^ the hydrodynamic volume of the MNPs in hexane was assumed to be that of the core of diameter 〈d〉 plus an oleic acid surface layer of thickness δ = 2.0 nm as estimated from DLS analysis (therefore, V_h_ = π(d + δ)^3^/6; see Table S1 in the Supplementary Information). The calculations were done for magnetic field amplitudes H_0_ such as 7.9 × 10^−2^ ≤ *H*_0_ ≤ 38 kA/m and frequencies *f* such as 10^−1^ kHz ≤ *f* ≤ 2 × 10^5^ kHz.

**Figure 1:**
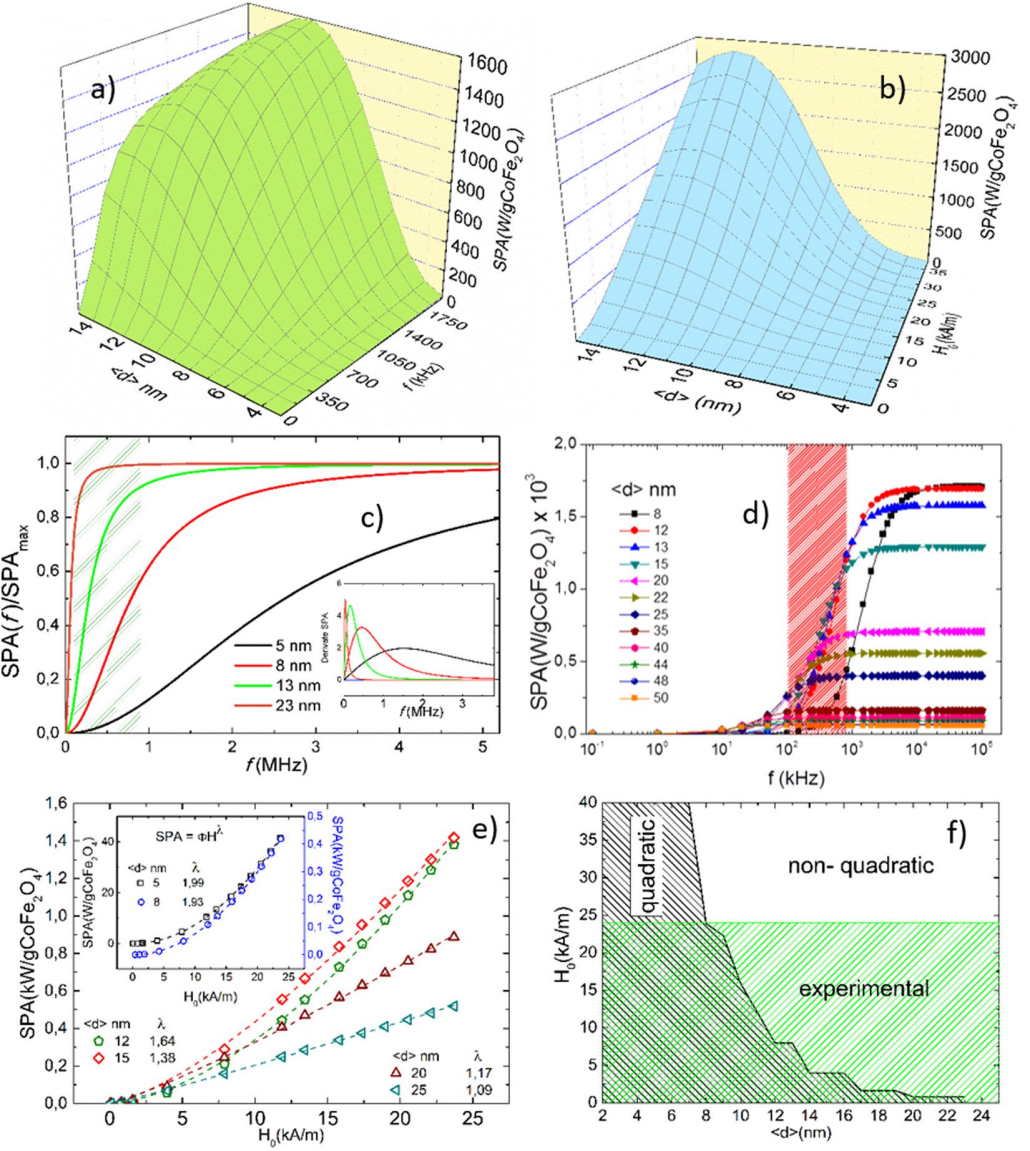
Results of the numerical simulations using Eq. 4 of (*a*) SPA vs. particle diameter and frequency (H_0_ = 18.5 kA/m). (**b**) SPA vs. particle diameter and H_0_ (f = 580 kHz). (**c**) SPA vs. frequency and particle diameter obtained with Eq. (5) for ΓHo^2^ ≈ 1 condition. The shaded area corresponds to the experimental frequency range (250–828 kHz) in this work. The inset shows the derivative of the SPA curves. (**d**) Semi-logarithmic representation of SPA vs. f at (H_0_ = 18.5 kA/m) for particle diameters 8 < d < 50 nm (solid symbols are calculated data; lines are a visual guide). The shaded area indicates the experimental frequency window in this work. (**e**) SPA vs. H_0_ (*f* =  580 kHz) for particle sizes 5 ≤ d ≤ 25 nm. Dashed lines represent the best fit using the power law ${SPA}\cong {\Phi H}^{\lambda }$. The line is a visual guide. (**f**) H_0_ vs. 〈d〉 (*f* = 580 kHz) plot. The black shaded area delimits the particle size values for which the condition $SPA\propto {{\rm{H}}}_{0}^{2}$ is fulfilled. The green shaded area identifies the (H_0_, d) space of experimental fields H_0_ ≤ 24 kA/m of this work. All simulations were performed with M_S_ = 4.2 × 10^5^ A/m, *K*_*eff*_ = 2 × 10^5^ J/m^3^, and η = 2.94 × 10^−4^ kg/ms (see text).

Figure 1 presents the resulting simulations of the SPA as a function of the frequency when H_0_ = 18.5 kA/m (Fig. 1a), and as a function of the field amplitude when *f* = 580 kHz (Fig. 1b). A common feature of these curves is that for some 〈d〉 values, the SPA has a maximum. The size that maximizes the SPA depends on the frequency and, to a less extent, on the magnetic field intensity H_0_. Some of the values presented in the Fig. 1a,b are beyond the limit of the LRT in our case because (*k*_*B*_*T* < *μ*_0_*M*_*S*_*VH*_0_), Nevertheless, we performed such simulations because they allow us to understand how is the variation of SPA with H, *f* and d within a range of reasonable experimental values.

To address these dependences separately, we will explore first the shape of the frequency dependence of the SPA, assuming in this case the expansion of the Langevin function around H_0_ ≈ 0, so that the susceptibility of the system is *χ*_0_ = *χ*_*i*_ (i.e., the initial susceptibility, which is by definition a field-independent parameter; see the Supplementary Information for details. According to (4) the SPA (H_0_, *f*) has a simple dependence:

5 $$SPA={{\rm{\Gamma }}H}_{0}^{2}\frac{{\rm{B}}{f}^{2}}{{({\rm{B}}f)}^{2}+1}$$

where Γ is a field- and frequency-independent parameter that contains the magnetic properties of the MNPs, and B = 2πτ_*eff*_. Figure 1c shows the frequency dependence of the SPA for the ΓHo^2^ ≈ 1 condition, calculated by Eq. (5) for four different particle sizes (i.e., for four different values of τ_*eff*_ in B). There is an upper limit for the SPA, whose value are given by Γ (i.e., characteristic of each particle). The green shaded area in Fig. 1c comprises the most common frequency values reported in SPA measurements, coinciding with the experimental frequency range in this work and includes the points where the SPA has the steepest changes only for particles with *d* ≥ 8 nm. The inset of Fig. 1c shows that the particle size with maximum derivative shifts above *f* ≥ 1 *Mhz* values for *d* ≤ 8 nm and, for MNPs larger than ≈20 nm the maximum SPA is already attained at ≈50 kHz. Figure 1d shows a semi-logarithmic representation of the frequency dependence of the SPA calculated from Eq. (4) for MNPs with 〈d〉 up to 50 nm (i.e., below the critical single-domain diameter reported for cobalt ferrites)^10^.

In this case, all curves have similar dependences: almost no power absorption is observed for *f* < 10^1^ kHz, which is followed by a steep increase for frequencies such as ${10}^{1}\lesssim f\lesssim {10}^{3}$ kHz, and a saturation of the SPA for *f* > 10^3^ kHz. The shaded area in the Fig. 1d shows the frequencies values used in our experiments and in most of the experiments found in the literature. According to the figure, as the particle diameter increases, the frequency for which the maximum increase of the SPA is observed shifts to lower values. Whereas for diameters around 3–4 nm this region is centered at *f* ≈ 100 MHz, it shifts to 30–60 MHz for 〈d〉 between 13 and 25 nm. For 〈d〉 ≥ 35 nm the SPA is already saturated at *f* ≈ 5 MHz.

Figure 1e shows the SPA dependence on H_0_ for *f* = 580 kHz calculated with Eq. 4. Assuming χ_0_ is field-dependent (See Eq. S10). The results show that despite the system fulfills the condition H_0_ ≫ H_K_, a deviation of the quadratic dependence of SPA with the magnetic field strength H_0_ is observed. This deviation could be due to the magnetic field dependence of τ_B,_ as was reported by Yoshida and Enpuku^11^ and was not taken into account in our model.

These results were fitted with a power law *SPA* = Φ*H*^*λ*^, with λ between 1.99 and 1.09. Our results show that at fixed frequency, the quadratic field dependence of the SPA is only fulfilled up to certain values of the magnetic field strength that depend on the particle size. For increasing particle size the maximum magnetic field for which $SPA\propto {{\rm{H}}}_{0}^{2}$ holds decreases. In particular, at *f* = 580 kHz, the quadratic dependence for fields H_0_ ≤ 24 kA/m (the experimental maximum value in this work) is satisfied only for the particles with d ≤ 8 nm, expected in a certain way due to the argument mentioned above, (*k*_*B*_*T* < *μ*_0_*M*_*S*_*VH*_0_). This region of experimentally achieved fields is represented in Fig. 1f as the green shaded area labeled “experimental”, which defines the attainable (H_0_, d) values in our experiments. The black shaded area labeled ‘quadratic’ represents the expected (H_0_, d) loci for which the $SPA\propto {{\rm{H}}}_{0}^{2}$ dependence is fulfilled, calculated for fields up to 40 kA/m. The diagram shows that only cobalt ferrite particles with 〈d〉 ≤ 8 nm are expected to obey this quadratic dependence for any applied field intensity. On the other hand, for those particles with 〈d〉 ≥ 25 nm the $SPA\propto {{\rm{H}}}_{0}^{2}$ condition should never be expected. We understand that this model is not the more rigorous but it is an easy tool in order to explore the frequency and amplitude field dependence of the SPA for systems where the mechanical mechanism of magnetic relaxation is dominate.

### Comparison to colloids and *in vitro* experiments

**Figure 2:**
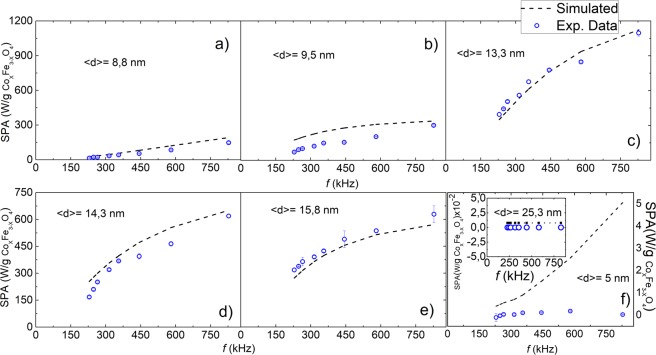
SPA vs. frequency: the experimental data (blue circles) are obtained at H_0_ = 18.5 kA/m. Dashed lines are the SPA resulting from the simulations; the respective input parameters are obtained from the respective sample physicochemical characterization (see text).

**Figure 3:**
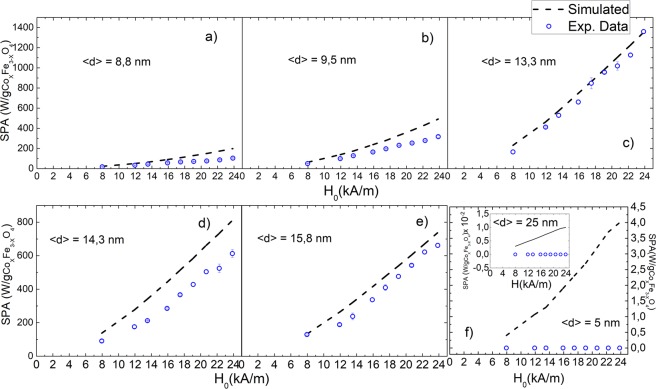
SPA vs applied magnetic field intensity H_0_: the experimental data (red circles) were obtained at *f* = 580 kHz. Dashed lines are the SPA resulting from the simulations; the respective input parameters were obtained from the respective sample physicochemical characterization (see text).

The SPA of seven samples of Co-ferrite MNPs in hexane with average particle diameters 5 ≤ 〈d〉 ≤ 25 nm was measured at frequencies 229 ≤ *f* ≤ 828 kHz and magnetic field amplitudes 9.5 ≤ H_0_ ≤ 24 kA/m (see materials and methods section). The experimental values were compared to the numerical simulations. For each sample, the input parameters for the simulations were those obtained from the respective structural and magnetic characterization of the MNPs (size, size distribution width, hydrodynamic radius, anisotropy constant, and saturation magnetization), previously reported elsewhere^8^. The anisotropy constants *K*_eff_ and saturation magnetization M_S_ of these samples were found to be within 1.2 × 10^5^ < *K*_eff_ < 3.78 × 10^5^ J/m^3^, and 24 ≤ M_S_ ≤ 76 Am^2^/kg, respectively. The corresponding anisotropy fields H_K_ estimated from these numbers (1.2 × 10^3^ < H_K_ < 1.9 × 10^3^ kA/m) satisfy the condition H_0_ ≪ H_K_ in the whole range of experimental applied fields. The comparison between numerical calculations and the experimental data are shown in Figs 2 and 3.

A reasonable agreement between the simulated and experimental values of the SPA was achieved for the whole series of samples, except for the 〈d〉 = 5 nm sample for the reasons discussed below. The agreement between simulations and experiment for 〈d〉 = 13 nm, i.e. the size of the maximum SPA, is remarkable. For those samples with intermediate SPA values the differences could be assigned to partial agglomeration of the MNPs in the suspension that yields an underestimation of the hydrodynamic diameter and affects the calculated τ_B_ (see Table S1 in Supplementary Information). The large differences between the calculated and experimental SPA for the sample with 〈d〉 = 5 nm (5 W/g and 0 W/g respectively, at the maximum field) are likely to be originated in the spin surface configuration of this sample. Indeed, the magnetic characterization of this sample showed that the magnetization was largely diminished in comparison to the other samples, and did not saturate even for applied fields larger than 11.2 × 10^6^ A/m. Also, the M(H) curves for this small MNPs showed an open hysteresis loop up to that maximum attainable field^8^. These observations are consistent with a spin canted configuration of the surface magnetic moments, a well-known effect for small-sized ferrite nanoparticles^12,13^. Moreover for these particles the corresponding Néel and Brown relaxation times have similar values (see Fig. S3 in Supplementary Information) and therefore the two mechanisms cannot be considered as independent. A more realistic analysis of this situation would require numerical simulations using the comprehensive models proposed by N.A. Usov *et al*. and H. Mamiya *et al*.^4–6^.

We note also that the relaxation mechanisms depend also on the applied magnetic field, as reported by Yoshida and Enpuku^11^. According to these authors, nonlinear effects can be expected in Brown relaxation at high applied magnetic fields, which is supposed to be more significant for systems driven by Brown relaxation, as is the case in the Co-ferrite MNPs studied in this work. Accordingly, when analyzing the dependence of the SPA with applied fields (Fig. 3) up to H_0_ = 24 kA/m (a value 20% larger than that in Fig. 2) the discrepancies easier to see, suggesting that nonlinear effects in τ_B_ are operative. Since the values of SPA calculated numerically for particles with 〈d〉 = 25 nm are of the order of ≈0.01 W/g, these values are well below our experimental resolution (≈2 W/g for the conditions of this sample), and therefore both sets of data are considered as equivalent.

In a recent work on Fe_3_O_4_ MNPs within the LRT framework^14^ it was shown that dipolar interactions due to agglomeration lower the SPA. Similar conclusions were drawn by Branquinho et al. in the case of MnFe_2_O_4_ MNPs^15^. Since our model did not include magnetic dipolar interactions, they could partially explain the discrepancies with the experimental SPA in Figs 2 and 3. Interestingly, there are also works attributing a SPA increase to agglomeration effects in MgFe_2_O_4_ and NiFe_2_O_4_ MNPs^16^. As mentioned in previous sections, in our samples the main mechanism involved is the Brownian relaxation, except in the case of the smallest particle volumes. As shown in Fig. S3, for the smallest particles, Néel and Brown relaxation times have similar values and therefore the Néel component in the power absorption should be also taken into account. In these cases, the mechanism of relaxation is more vulnerable to the dipolar interactions and the LRT show limitations for a complete description of the physical phenomenon i.e., the Specific Power Absorption.

The limitations of the LRT framework to account for dipolar interactions have been discussed previously^15,17^ and there is consensus on the need to use more realistic models^18^ as well as to precisely characterize the main magnetic and physicochemical parameters of each specific magnetic colloid^19^. Values of SPA in Co-ferrite MNPs with 〈d〉 between 6 and 41 nm have been reported to range between 6 W/g and 800 W/g^20–27^. Since these values have been obtained at different H_0_ and *f* and being the MNPs dispersed in different liquid carriers, the comparison with our SPA experimental values is not straightforward. The reported data show that at fixed H_0_ and *f* the maximum SPA is obtained for MNP diameters about 12–13 nm^24^. The systematic SPA measurements on a series of Co-ferrite MNPs performed by A Sathya *et al*.^28^ show the same size dependence, being the optimal size for the maximum SPA about 17 nm. In addition, a steep dropping of SPA values below and above the optimal MNP size has also been reported^22,26^.

As mentioned above, the viscosity η of the carrier media determines the effective value of SPA when Brownian relaxation is the dominant mechanism^28,29^. Our simulations of the SPA *vs*. 〈d〉 curves in carrier liquids with different viscosities (water, hexane and blood) displayed the expected drop in SPA for increasing η values, keeping the bell-shaped dependence with a maximum at some optimal size value 〈d〉_op_ (see Fig. S9 in the Supplementary Information). The maximum of SPA shifts to lower values of 〈d〉_op_ for increasing viscosities (Fig. S9a), underlining the relevance of knowing the actual environment where the MNPs are going to be used. For magnetic hyperthermia, this implies to characterize the biological media where the heating by the MNPs is required^30,31^.

In the following paragraphs of this section, all the *in vitro* experiments discussed have been performed using a sample of Co-ferrite MNPs specifically chosen because of its high SPA (≈570 W/g in hexane). The crystallographic structure and magnetic data for this sample are shown in Fig. S2 of the Supplementary Information. The MNPs were transferred to aqueous suspension following the procedure described in the material and methods section. The study of the *in vitro* heating efficiency was performed on human neuroblastoma SH-SY5Y cells. The cell viability after incubation with the MNPs as a function of the concentration (see Figs S10 and S11 on Supplementary Information) was consistent with previously reported data on SH-SY5Y cells using Fe_3_O_4_ particles^32^. However, we mention here that due to the positive surface charge of the magnetite MNPs the effective amount bonded/incorporated to the cells was much larger in ref.^32^. In the present case, the intracellular distribution of MNPs after overnight incubation was scrutinised by Transmission Electron Microscopy (TEM) and Focused Ion Beam - Scanning Electron Microscopy (FIB-SEM).

**Figure 4:**
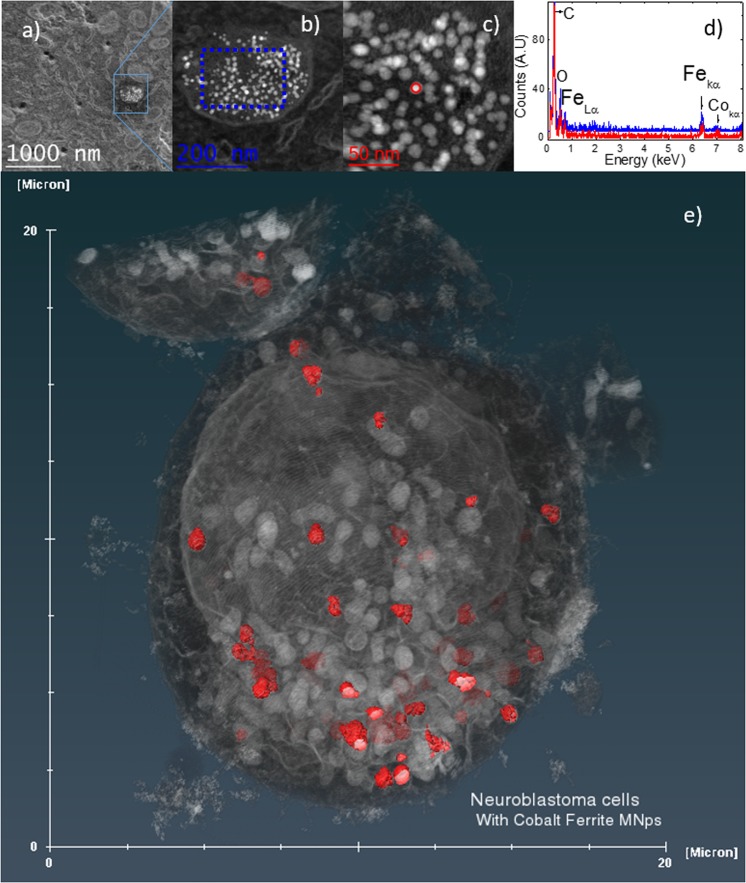
(**a**) STEM image of SH-SY5Y cells incubated with Co-ferrite nanoparticles at 20 µgml^−1^ for 24 hours. (**b**,**c**) Are zooms of the region selected on (**a**). (**d**) EDS spectrum (in blue) of the small group of MNPs selected in (**b**) and EDS spectrum (in red) of the particle selected in (**c**). Both show the presence of iron and cobalt in the MNPs. (**e**) Snapshot of the 3D cell reconstruction incubated with Co-ferrite MNPs at 100 µgml^−1^ for 24 hours. Red spots correspond to MNP aggregates.

For all the MNP concentrations studied, the images showed similar general trends: after proper washing of the culture, most of the remaining MNPs were located within the cytoplasm, specifically within vesicles, as small aggregates. A minor amount of MNPs attached to the cell membrane were also observed. The images in Fig. 4 correspond to a single cell incubated with 20 µg/ml of MNPs (Fig. 4a). Figure 4d shows the Energy Dispersive X-ray spectroscopy (EDS spectra) of a MNP cluster inside a vesicle (Fig. 4b) and of a single particle (Fig. 4c). These EDS–HAADF (High Angle Angular Dark Field) spectra showed the Kα and Lα peaks expected for iron and cobalt, characteristic of the composition of our nanoparticles. Moreover, it can be observed that the morphology of the MNPs is preserved inside the vesicles, ruling out any significant particle degradation (see also the Supplementary Information). The 3-D reconstruction of several cells (Videos VS1 and VS2 in the Supplementary Information) suggests an endosome-mediated uptake process.

**Figure 5:**
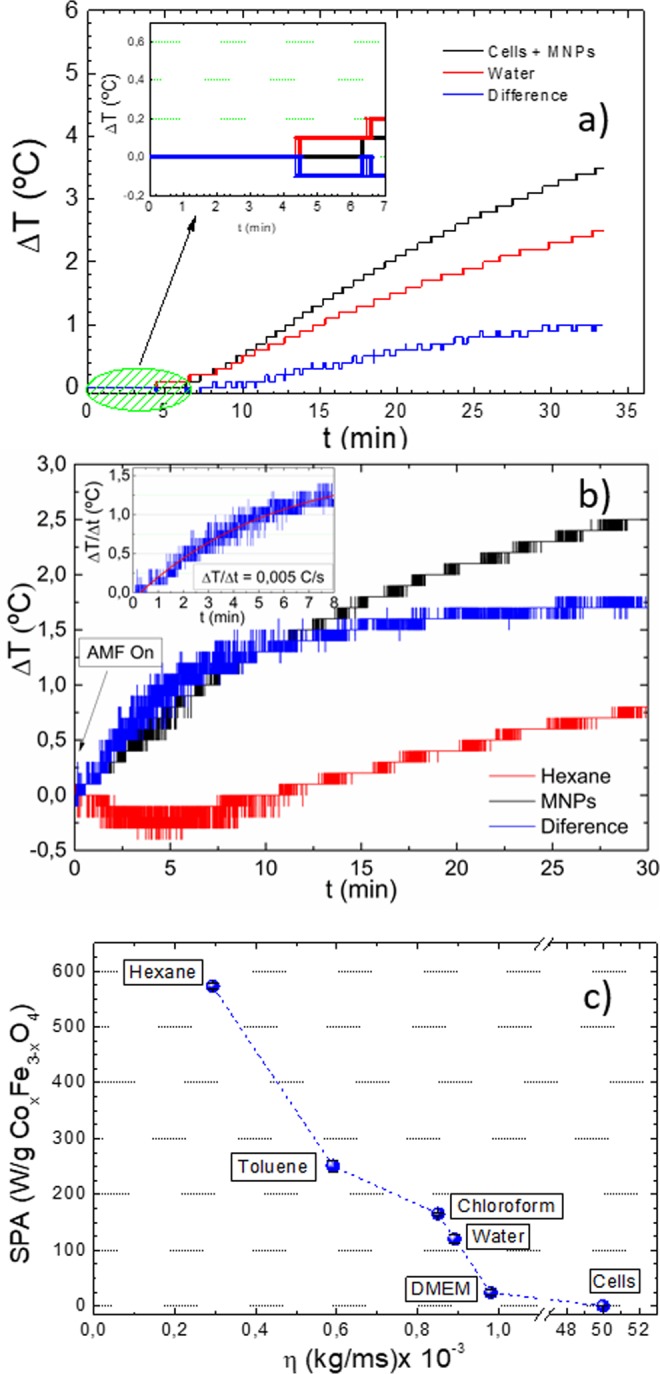
(**a**) Heating curves of SH-SY5Y cells loaded with MNPs (black curve) and of 1 ml of pure water (red curve) as control sample. The blue curve is the difference between both experiments. Inset: magnification of the same curves for t ≤ 7 min. (The applied field was turned on at t = 0). (**b**) Heating curves of the as prepared MNPs sample diluted up to 2 × 10^−2^ mg/ml in hexane (black); pure hexane (red) and the difference between both curves (blue). Inset: heating curves with a modified Box-Lucas fit. (**c**) SPA of the MNPs dispersed in different media as a function of the solvent viscosity, included the cell culture medium DMEM (Dulbecco modified Eagles minimal essential medium). All the experiment were carried out at H_0_ = 24 kA/m and *f* = 571 kHz.

For the power absorption experiments, the cells were incubated with the maximum MNP concentration for which a negligible cytotoxicity was observed (100 μg/ml), corresponding to a MNP concentration of 2 × 10^−2^ mg/ml inside the cells after incubation. The variation of temperature observed during the power absorption experiments is represented in Fig. 5a together with the data corresponding to a control sample (1 ml of pure water). The water curve represents the background signal arising from the self-heating of the equipment (from the Joule heating of the coil), and it was measured to subtract it from the heating data of the samples. The inset of Fig. 5a clearly shows that during the first ≈5–7 minutes no temperature increase was observed in the sample containing MNP-loaded cells. Moreover, after subtracting the control curve the maximum temperature increase measured after 2100 s was ΔT ≤ 0.9 °C, much lower than expected from the SPA calculations/measurements on the as synthesized MNPs. The small temperature increase observed is consistent with the distribution of the MNPs inside the cytoplasm, i.e., forming large agglomerates within vesicles. Together with the high viscosity of this environment it results in a large increase of the Brown relaxation time, ${\tau }_{B}=\frac{3\eta {V}_{h}}{{k}_{B}T}$, thus hindering viscous losses. To verify that the nearly-zero temperature increase was due to the blocking of the MNP rotation and not to a low MNP concentration inside the cell, we dispersed 2 × 10^−2^ mg/ml of MNPs in hexane. We measured the temperature increase ΔT in this sample, and subtracted the background heating using pure hexane as a control. The heating curves are shown in Fig. 5b.

There is a marked difference between the heating profiles of the control sample and the MNPs colloid, even shortly after starting the measurement (t < 600 s). A fast temperature increase is observed for the MNPs sample (about 1.5 °C in the first minutes). We calculated the SPA of the MNPs sample after background subtraction, by fitting the difference curve for t < 400 s, using a Box-Lucas function^33^, obtaining ΔT/Δt = 0.005 °C/s, which produces an SPA = 367 W/g. This value is 35% lower than that of the *as synthesized* sample, probably because the measured ΔTs are within the resolution limit of our equipment at the experimental conditions described. These results rule out the low concentration as the reason for the absence of heating of the *in vitro* experiments. The effect of the liquid viscosity on the experimental SPA values was assessed by measuring the same MNPs in different solvents (hexane, toluene, chloroform, water and cell culture medium, see Fig. 5c). The actual intracellular viscosity used has been assumed to be η = 5 × 10^−2^ kg/ms as reported in ref. ^34^. The decrease of SPA with the increasing viscosity confirms the major contribution from Brown relaxation to the heating mechanism.

## Conclusions

Our investigation on the heating capability of highly anisotropic Co-ferrite nanoparticles confirmed that the SPA values up to ≈1300–1400 W/g obtained in low-viscosity media originate in a purely Brownian relaxation mechanism. The numerical simulations provided a good match of the power absorption with the systematic experimental data. Deviations of the $SPA\propto {{\rm{H}}}_{0}^{2}$ dependence of the SPA with the applied field expected within the LRT emerged for certain values of H_0_ and *f* that depend on the average particle size considered. *In vitro* SPA measurements confirmed the total suppression of the Brown relaxation at the intracellular level, and the intracellular distribution of the MNPs within vesicles (either endosomes or lysosomes) provides further support to the hypothesis that the stalling of the Brown relaxation is due to a high viscosity local environment.

## Materials and Methods

### Synthesis of Co-ferrite nanoparticles

The cobalt ferrite nanoparticles used here were synthesized by thermal decomposition of iron acetylacetonate (acac)_3_ and cobalt acetylacetonate (acac)_2_ in organic solvents and in the presence of oleic acid and oleylamine^35,36^ as surfactants. Different organic solvents (phenyl ether, benzylether, 1-octadecene, and trioctylamine) having different boiling temperatures, were used in order to control the final particle size. The details about the preparation were reported in a previous work^8^. The method used for their stabilization in water was the interspersion of an amphiphilic polymer with the oleic acid on the nanoparticle surface. The general diagram for the transfer process is the following: the hydrophobic part of the polymer, the monomeric unit of 1-octadecene, has lateral chains of 18 carbon atoms that are insert with the oleic acid interacting hydrophobically. The hydrophilic part, the monomer unit of maleic anhydride, is exposed outside and is capable to stabilize the nanoparticles in aqueous medium by the carboxyl groups that are generated by hydrolysis of the anhydrides (made using sodium hydroxide)^37^. For the present work it is important to mention that, down to the single-particle analysis, all samples consisted of an homogeneous phase Co_x_Fe_3−x_O_4_, with a systematic deviation from the stoichiometric x = 1. The histograms for all samples showed small size distribution width (*w* = 2.9), exemplified with samples AV09 (〈d〉 = 9.5 nm) and AV15 (〈d〉 = 15.0 nm) in Fig. S1 of Supplementary Information.

### Transmission Electron Microscopy (TEM)

The detailed structural and morphological characterization of the samples studied in this work was carried out by transmission electron microscopy TEM and STEM modes. The TEM images were obtained using a thermoionic LaB_6_ 200 kV Tecnai T20 microscope (FEI Company) operating at an accelerating voltage of 200 kV. STEM–HAADF images were acquired using an XFEG TITAN 60–300 kV (FEI Company), operated at 300 kV, equipped with a monochromator and a probe aberration corrector (CEOS). For these experiments a drop of the MNP suspension (either in hexane or in water) was deposited on a holey carbon coated micro-grid. TEM images were obtained after the evaporation of the solvent, when the sample dried completely. From these images, size histograms were obtained. They were fitted using a Gaussian distribution function *g*(*x*)) of parameters (*w*, *d*_0_) given by:

6 $$g({d}_{0})=\frac{1}{w\sqrt{\frac{\pi }{2}}}ex{p}^{\lfloor -2{(\frac{d-{d}_{0}}{w})}^{2}\rfloor }$$

being *w* and *d*_0_ the FWHM (full-width at half-maximum) and the mean value of the distribution, respectively.

### Magnetic Characterization

Magnetization measurements M(H) were performed on a commercial SQUID magnetometer (MPMSXL Quantum Design) on dried samples. The powder was conditioned inside plastic capsules as described elsewhere^8^. Magnetic measurements for quantification of the cellular MNP uptake were done at room temperature in a vibrating sample magnetometer (Lake Shore 7400 Series VSM), as a function of the field up to 1.5 T.

### Heating Efficiency Measurements

Power absorption experiments under ac magnetic fields were performed in a commercial applicator (DM100 from nB nanoscale Biomagnetics, Spain) at frequencies such as 229 ≤ *f* ≤ 828 kHz and applied magnetic fields such as 7.95 ≤ H_0_ ≤ 24 kA/m. The SPA values of magnetic colloids containing a total mass m_NP_ of MNPs dispersed in a mass m_l_ of carrier liquid were calculated as:

7 $$SPA=\frac{P}{{m}_{NP}}=\frac{{m}_{l}{c}_{l}+{m}_{NP}{c}_{NP}}{{m}_{NP}}\,(\frac{{\rm{\Delta }}T}{{\rm{\Delta }}t})$$

where *c*_*l*_ and *c*_*NP*_ are the specific heat capacities of the liquid carrier and the magnetic nanoparticles, respectively. ΔT is the temperature increase of the sample measured in a time interval Δt. Since for these experiments the concentration of MNPs is usually in the range of 1% wt., we can approximate ${m}_{l}{c}_{l}\,+{m}_{NP}\,{c}_{NP}s\approx {m}_{l}{c}_{l}$ and the Eq. (7) can be written as

8 $$SPA=\frac{{C}_{l}{\delta }_{l}}{\varphi }\,(\frac{{\rm{\Delta }}T}{{\rm{\Delta }}t})$$

where δ_*l*_ and ϕ are the density of the liquid and the (mass) concentration of the MNPs in the colloid, respectively. The heating rate ΔT/Δt in °C s^−1^ is obtained from the initial temperature increase, within the first 50–100 seconds of the experiment.

### Dynamic Light Scattering

The distribution of hydrodynamic diameters when the nanoparticles are dispersed in hexane or in water was obtained using a Brookhaven Instruments 90 Plus photon-correlation spectrometer.

### MNPs Cell culture

Dulbecco’s modified Eagle’s medium (DMEM) was chosen as the medium for culturing human neuroblastoma SH-SY5Y cells (ATCC CRL-2266), in a mixture of Ham’s F12 (1:1) mixture with 15% fetal bovine serum. Aliquots of penicillin (100 IU/ml) and Streptomycin (100 µg/ml) were added to the culture medium together with 2 mM of L-glutamine. Incubation of the cells was done in all cases maintaining the samples at 37 °C within an incubation atmosphere composed by 95% air and 5% CO_2_. The SPA experiments in cell pellets were performed after the cells were incubated for 24 hours with different concentrations of MNPs, then washed several times and the culture medium was replaced by fresh, ordinary DMEM composition medium. Cells for control samples (i.e., without MNPs) were grown simultaneously in each experiment, in order to replicate possible environmental fluctuations from pellet to pellet.

### Cell Viability Assays

Cell viability assays were performed after incubation with MNPs using ≈80 × 10^3^ cells at the exponential growth phase of the cell cycle. The cells were seeded onto a twelve-well plate and incubated for 24 h. After that, culture medium with increasing MNPs concentrations (1, 5, 10, 20, 50 and 100 µg/ml) was used to replace the modified-DMEM and the cells were incubated for another 24 h. Trypan blue assays were conducted by diluting 20 ml of cell samples into Trypan blue (1:1) before the viable cells were counted. The fractions of viable cells as compared to control cells were calculated, assuming 100% viability for the control cells. For flow cytometry analysis Annexin-binding buffer (composed by 5 ml of Annexin and 5 ml of propidium iodide) was used to incubate the cells for 15 min at room temperature, keeping a dark environment. The resulting viability was analyzed using a FACS Aria Cytometer and FACS Diva Software.

For the study of the intracellular MNPs distribution by TEM and FIB-SEM the cells were seeded (1 × 10^6^ cells/well) in a 6-well-plate in 2 ml of culture media. Following the protocol described above, after 24 h increasing concentrations of MNPs were added and the cells incubated overnight. After incubation, the cells were washed, detached and fixed with 2% glutaraldehyde solution for 2 h at 4 °C. They were washed again three times in cacodylate buffer (pH 7.2) and treated with potassium ferrocyanide 2.5% and osmium tetroxide 1% for 1 hour at room temperature. The cells were then dehydrated with increasing concentrations of acetone 30% (x2), 50% (x2), 70% (x2), 90% (x2), and a final dehydration with pure acetone. After drying, samples were embedded in a solution (50:50) of EPOXI resin and acetone (100%) overnight, and then for 4–5 hours in resin EPOXI 100%. These samples were maintained for 2 days at 60 °C.

### Dual Beam FIB-SEM Analysis

Intracellular distribution of MNPs was assessed using a dual beam FIB/SEM (Nova NanoLab 200 and Helios 650 Thermo Fisher Scientific). For these experiments the SH-SY5Y neuroblastoma samples were conditioned. SEM images were taken at 2–5 kV with a FEG column, and a combined Ga-based 30 kV (10 pA and 0.27–9 nA) ion beam was used to cross-sectioning single cells and resin pellet. Energy-dispersive x-ray (EDS) spectra were acquired in order to identify the presence of characteristic metal elements (iron and cobalt) in the MNPs.

## Supplementary information

Video VS1

Video VS2

Supplementary Information
