# Supplementary material for: The relevance of Brownian relaxation as power absorption mechanism in Magnetic Hyperthermia
Source: Sci Rep. 2019 Mar 8;9:3992. doi: 10.1038/s41598-019-40341-y (PMC6408542; doi:10.1038/s41598-019-40341-y)
Supplement: Supplementary file 3 — Supplementary Information [file 41598_2019_40341_MOESM3_ESM.pdf]

Supplementary Information

The relevance of Brownian relaxation as power absorption mechanism in Magnetic Hyperthermia.

Teobaldo E. Torres,* [1, 2] Enio Lima Jr., [3] M. Pilar Calatayud, [1] Beatriz Sanz, [1] Alfonso Ibarra, [1, 2] Rodrigo Fernández-Pacheco, [1, 2] Alvaro Mayoral, [1, 2] Clara Marquina, [4, 5] M. Ricardo Ibarra [1, 2, 4] and Gerardo F. Goya [1, 4]

[1] *Instituto de Nanociencia de Aragón (INA), Universidad de Zaragoza, C/Mariano Esquillor s/n, CP 50018, Zaragoza, Spain.*
[2]*Laboratorio de Microscopias Avanzadas (LMA), Universidad de Zaragoza, C/Mariano Esquillor s/n, CP 50018, Zaragoza, Spain.*
[3] *Div. Resonancias Magnéticas, Centro Atómico de Bariloche/CONICET, S.C 8400, Bariloche Argentina.*
[4]*Departamento de Física de la Materia Condensada, Facultad de Ciencias, Universidad de Zaragoza, Zaragoza Spain.*
[5] *Instituto de Ciencia de Materiales de Aragón (ICMA), Consejo Superior de Investigaciones Científicas (CSIC) - Universidad de Zaragoza, Zaragoza, Spain.*

† To whom correspondence should be addressed. E-mail: teo@unizar.es, Phone: (+34) 976762864, Fax: (+34) 976762777. KEYWORDS Magnetism, magnetic anisotropy, hyperthermia

Supplementary Information 1

Physicochemical characterization of Co-ferrite MNPs.

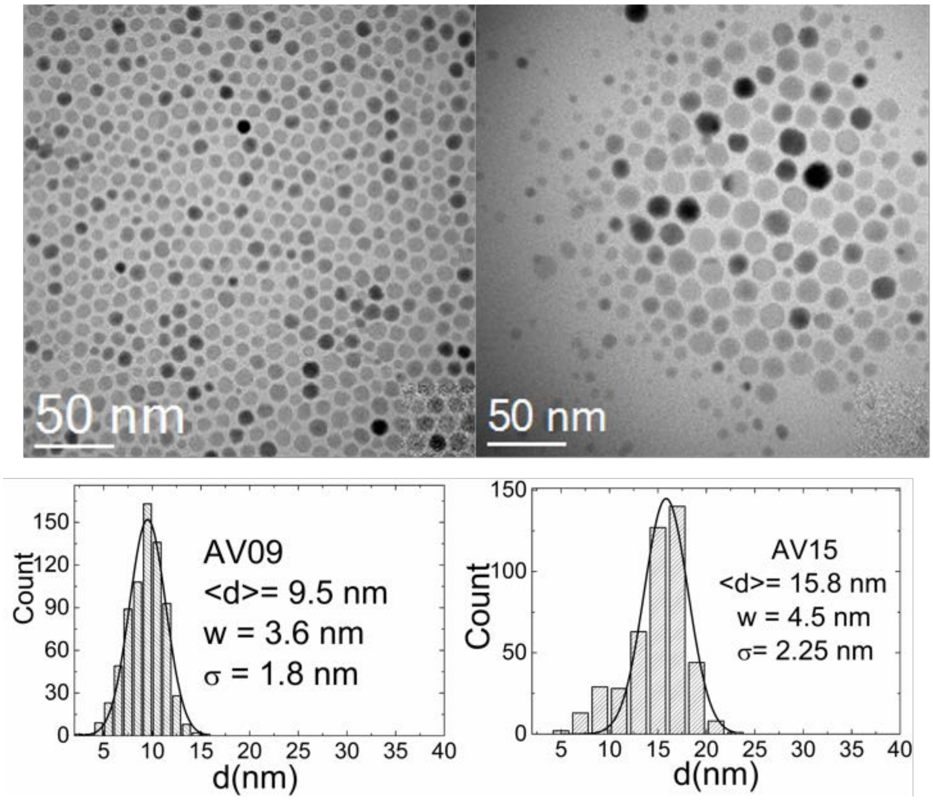

Figure S1: TEM images of cobalt ferrite nanoparticles in samples with diameters 9.5 nm (sample AV09, left panels) and 15.8 nm (sample AV15, right panels). The lower graphs represent the corresponding Gaussian size distributions. The parameters w and σ correspond to the size distribution width and standard deviation, respectively.

Their morphological and magnetic properties of all samples used in this work were similarly characterized[1].

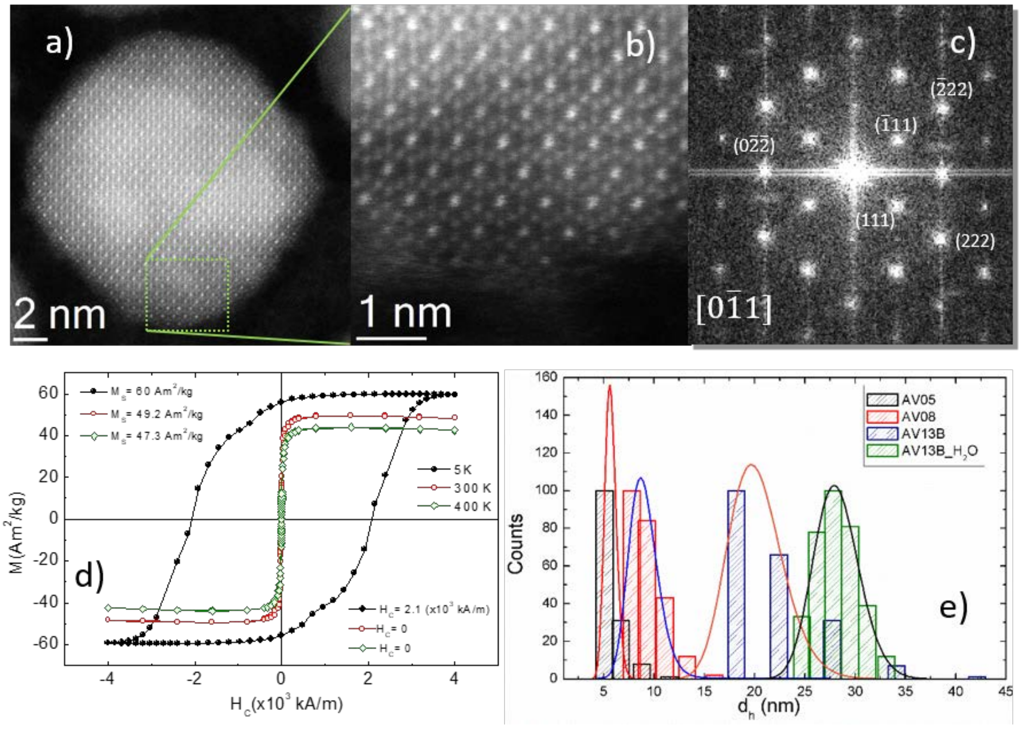

Figure S2 a,b and c) High resolution Cs-corrected STEM-HAADF image with the respective Fast Fourier Transform of sample with <d> = 13.9 nm (labelled as AV13B), used in *in vitro* experiments (cytotoxicity and MNP cellular uptake, intracellular distribution an *in vitro* power absorption studies) d) Hysteresis loops at different temperatures. e) Size histograms obtained from DLS measurements of samples dispersed in hexane with <d>= 5 and 8.8 nm (labelled as AV05, and AV08 respectively) and sample with <d> = 13 nm (labelled as AV13B) dispersed in hexane and water.

The atomic resolution images in Figure S2 reveal that the atomic periodicity corresponding to a spinel structure with no defects extends to the particle surface.[1] The average <d> and the hydrodynamic d_h diameters of MNPs dispersed in hexane and water (obtained by Dynamic Light Scattering) are shown in Table S1. The results show that, when suspended in hexane, all samples except AV25 have hydrodynamic sizes very similar to the diameter derived from the analysis of the TEM images by sampling individual nanoparticles. This fact suggests that the nanoparticles are well dispersed in this organic solvent. The similar values of $\langle d \rangle_{TEM}$ and d_h in hexane agree as well with the hypothesis that the oleic is attached to the MNPs surface forming a monolayer.

Table S1. Average diameter <d>$_{TEM}$, size distribution width (w) and size dispersion (σ_{TEM}) obtained from TEM images. Hydrodynamic diameters (d_h) in hexane and water and the respective size dispersions (σ_{hex} and σ_{wat}) have been obtained from DLS measurements.

Sample	<d>$_{TEM}$ (nm)	W_{TEM} (nm)	σ_{TEM} (nm)	Hexane d_h (nm)	Hexane σ_{Hex} (nm)	Water d_h (nm)	Water $\sigma_{Wat.}$ (nm)
AV05	5.5	1.48	0.8	5.7	0.4	17.8	1.1
AV08	8.8	2.6	1.3	8.9	1.4	15.6	1.1
AV09	9.5	3.2	1.8	12.3	1.5	65.5	5.8
AV13	13.3	2.6	1.3	15.3	0.9	48.9	3.4
AV13_B	13.9	5.8	2.9	20.1	1.2	28.1	2.2
AV14	14.3	3.2	2.6	16.1	0.7	30.6	3.2
AV15	15.8	4.6	2.3	16.1	1.4	29.5	2.9
AV25	25	4.2	2.1	800	32.5	*	

*Not available (the nanoparticles were not transferred to water)

The values of <d>$_{TEM}$ and d_h derived from the magnetic and structural characterization and displayed in Table S1are the input parameters for the calculation of the magnetic and hydrodynamic volumes ($V_M = \pi \frac{d^3}{6}$ and $V_h = \pi \frac{(d+2\delta)^3}{6}$, respectively) in the SPA numerical simulations. The thickness of the organic layer δ was assumed as 2 nm in all cases.

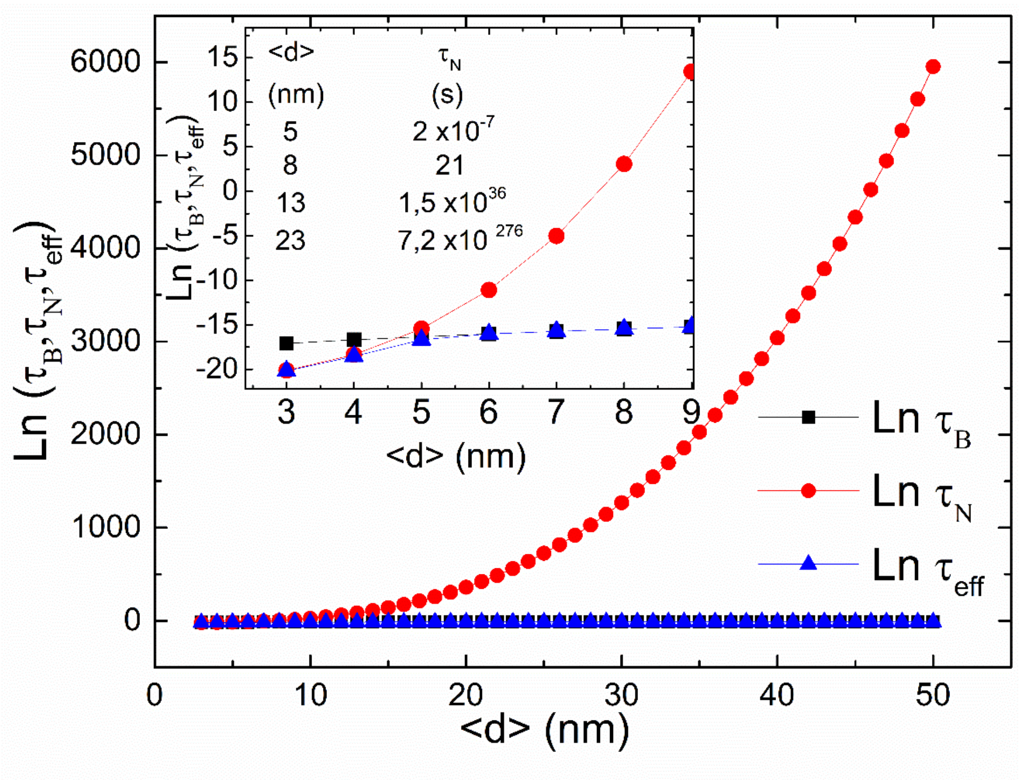

Figure S3 Brown, Neél and effective relaxation times for Co-ferrite MNPs of different average diemters <d>, assuming the viscosity of the hexane η= 2.94x10^{-4}, the magnetic anisotropy constant of the bulk phase K= 2x 10^5 J/m^3 and τ_0= 5x10^{-9} s. The calculations were done using equations (2) and (3) in the manuscript, with k_B = 1.38 x10^{-23} J/K and T= 300K.

Supplementary Information 2

SPA expression for the numerical simulations.

From thermodynamic considerations,[2] the variation of internal energy of a MNP ensemble that can be written as of function of the magnetic work as:

$$\Delta U = -\mu_0 \oint M dH \qquad \text{(S1)}$$

and the power is:

$$P = \frac{\Delta U}{T} = f\,\Delta U = -f\,\mu_0 \oint M dH \qquad \text{(S2)}$$

where T = 1/f is the period of *H(t)*. The SPA is the power by mass unit, also known as Specific Absorption Rate (SAR) or Specific Loss Power (SLP), given by:

$$SPA = \frac{P}{m}, \qquad \text{(S3)}$$

Assuming that the magnetic field is $H(t) = H_0\, cos(\omega t)$, the variation of internal energy can be write as:

$$\Delta U = \mu_0 {H_0}^2 \chi'' \pi \qquad \text{(S4)}$$

Therefore

$$P = f \mu_0 {H_0}^2 \chi'' \pi \qquad \text{(S5)}$$

with both components of magnetic susceptibility defined as:

$$\chi'' = \frac{\chi_0 \omega \tau}{(\omega\tau)^2 + 1} \qquad \text{(S6)}$$

$$\chi' = \frac{\chi_0}{(\omega\tau)^2 + 1} \qquad \text{(S7)}$$

Finally, substituting Eq. S6 in the expression of the power (Eq. S5), we obtain:

$$P = f {H_0}^2 \chi'' \pi = \mu_0 f {H_0}^2 \pi \frac{\chi_0 \omega \tau}{(\omega\tau)^2 + 1} \qquad \text{(S8)}$$

Using the expression of the angular frequency ω = 2πf, we obtain

$$P = \mu_0 f \pi H_0^2 \chi_0 \frac{2\pi f \tau}{(2\pi f \tau)^2 + 1} \qquad \text{(S9)}$$

where

$$\chi_0 = \chi_i \frac{3}{\zeta}\left(coth\zeta - \frac{1}{\zeta}\right) \qquad \text{(S10)}$$

is the susceptibility of an ensemble of magnetic nanoparticles in the equilibrium, with:

$$\zeta = \frac{\mu_0 M_S V_M H}{k_B T}; \quad \chi_i = \frac{\mu_0 V_M M_S^2}{3k_B T}; \quad \tau = \tau_{eff} = \frac{\tau_N + \tau_B}{\tau_N * \tau_B}\ ; \quad \tau_N = \tau_0\, e^{\frac{KV_M}{k_B T}} \ \text{ and } \ \tau_B = \frac{3\eta V_h}{k_B \mathrm{T}}$$

Where χ_i is the initial susceptibility and

$$V_M = \pi \frac{d^3}{6} \quad \text{and } V_h = \pi \frac{(d+2\delta)^3}{6}$$

In the general case χ_0 is field dependent because ζ is field dependent. However, these definitions imply that in the limit of the magnetic field strength H→ 0 we have $\zeta \rightarrow 0$, i.e. the magnetization becomes linear with H (regimen of validity of LRT). Therefore the equation S10 can be written as

$$\chi_0 = \chi_i = \frac{\mu_0 V_M M_S^2}{3k_B T}.$$

These expressions show that when H → 0 the parameter χ_0 remains field-independent. , This is the assumption to analyze the shape of the frequency dependence of the SPA in Fig. 1c on the manuscript. Equation S9 is obtained assuming a monodisperse particle system (i.e., the same particle size for the whole ensemble with a delta size distribution). To calculate the SPA in real systems it is necessary to include a particle size distribution, represented by a function $g(d)$. In that case Eq. S9 becomes

$$SPA(\langle d \rangle) = \int_0^\infty SPA(d)\, g\,(d) d\mathrm{d}\ ,$$

which in its explicit form is

$$SPA(\langle d_0 \rangle) = \int_0^\infty \mu_0 f \pi H_0^2 \chi_0 \frac{2\pi f \tau_{eff}}{\left(2\pi f \tau_{eff}\right)^2 + 1} \times \frac{1}{w\sqrt{\pi/2}} exp^{\left[-2\left(\frac{d-d_0}{w}\right)^2\right]} d\mathrm{d}$$

This equation (Eq (4) in the main text) can be solved numerically. For that, we used an algorithm written within Maple 10 software, a computer symbolic algebra code. The algorithm evaluates the SPA expression as a function of *d* between 0 and 100, with increasing values of <d_0> between 3 and 50 nm, which covers the experimental size range of our samples.

Supplementary Information 3

Magnetic field dependence of magnetization of representative samples below 300 Oe (24 kA/m).

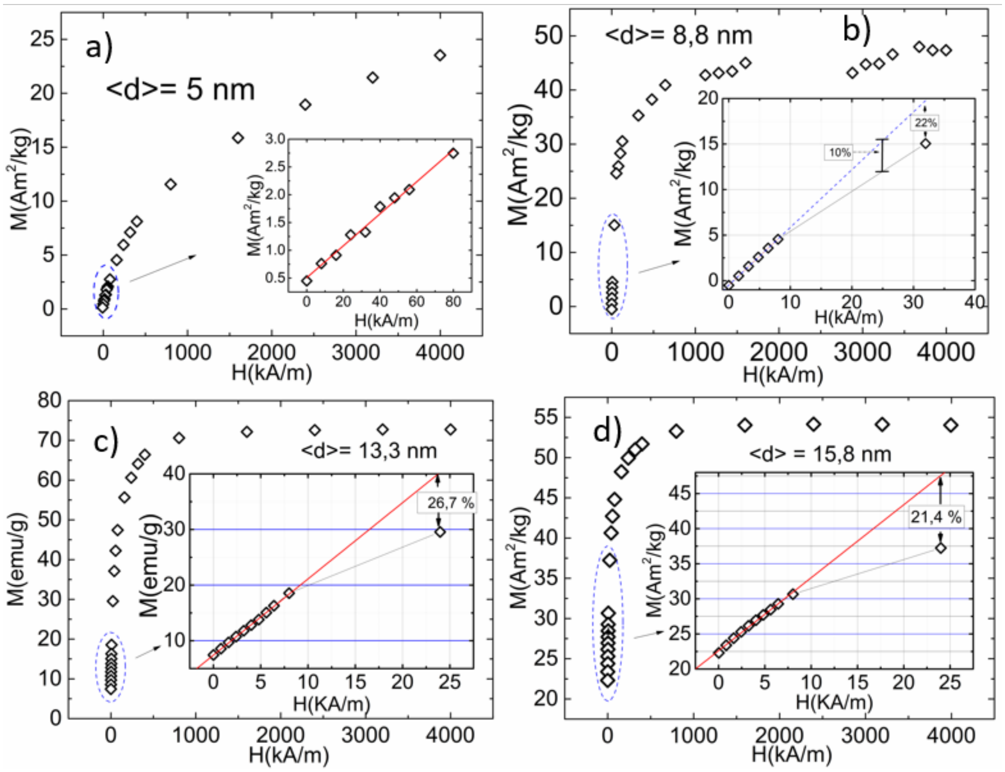

Figure S4. Room temperature M(H) curves up to H= 300 Oe. Deviations from the linear dependence about 10-27 % are observed at the maximum field, except for <d> = 5nm (panel a).

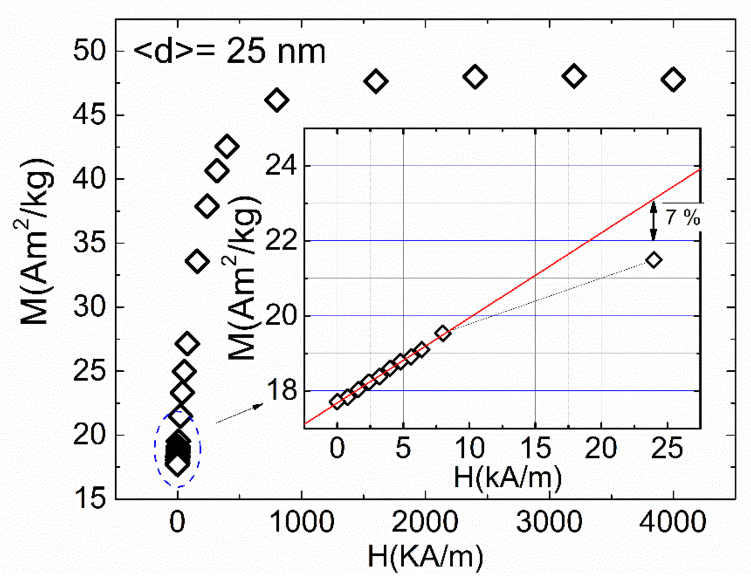

Figure S5 M(H) curve for the sample with <d>= 25 nm. A deviation from the linear behaviour of ≈7 % at H= 300 Oe is observed.

Supplementary Information 4

Simulations of the SPA as a function of the frequency, applied magnetic field and experimental results.

The dependence SPA $\propto$ H^2 must be fulfilled in the LRT limit and it is the clear signature for its validity in an applied magnetic field range. However, SPA vs H usually deviates from this quadratic behaviour. In the most general case, the SPA can be written as

$$SPA \cong AH^B \qquad \text{(S11)}$$

or

$$\log(SPA) = \log(A) + \mathrm{B} \cdot \log(H). \qquad \text{(S12)}$$

This relation represents a straight line when plotting log(SPA) *vs.* log(H), with B being the slope of the curve (B = 2 for the LRT limit). Figure S6 corresponds to a MNP sample with <d> = 3 nm and shows that the quadratic dependence of the SPA (see inset) is fulfilled for any magnetic field amplitude up to 159 kA/m (B=1,996).

Figure S7 shows the numerical simulations for several particle diameters. The maximum magnetic field for which the quadratic dependence of the SPA is fulfilled (marked by arrows in the Figure S7) decreases as the diameter increases. The values are shown in Fig. 1f in the manuscript.

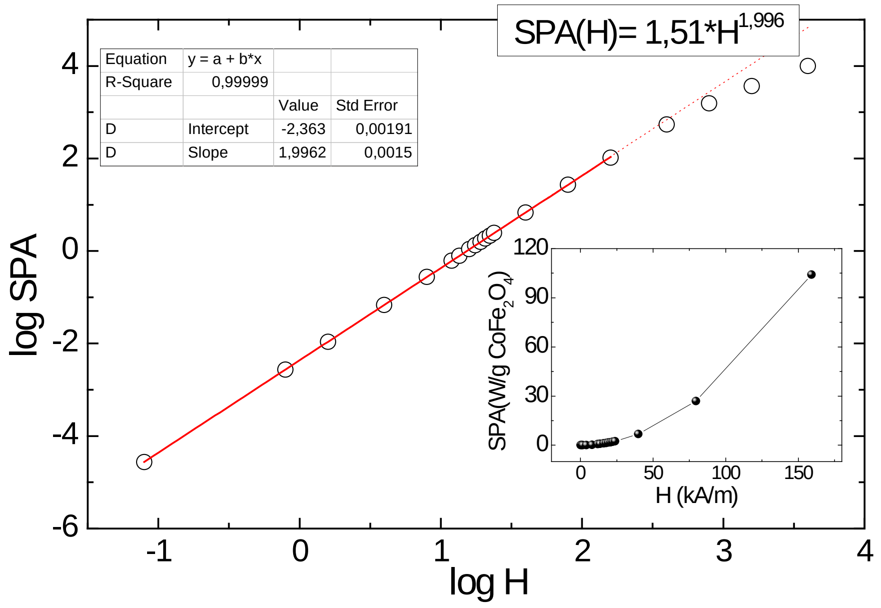

Figure S6: log(SPA) *vs.* log(H) obtained from numerical simulations for cobalt ferrite nanoparticles with <d> = 3 nm

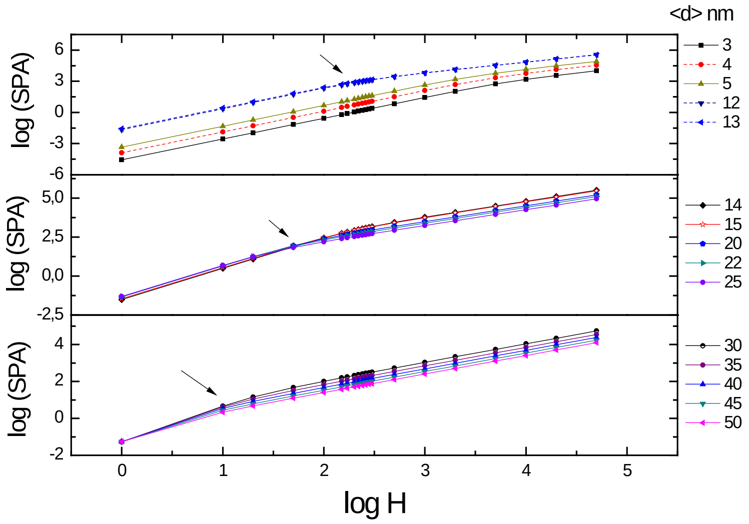

Figure S7 : log SPA vs log H, obtained from theoretical simulations for different particle diameters. Arrows show the maximum magnetic field amplitude for which the quadratic dependence of the SPA is fulfilled.

Figure S8 (a-b) shows the experimental SPA values as a function of the frequency and as a function of the magnetic field amplitude corresponding to the measurements on the synthesised samples dispersed in hexane. These values are collect in Table S2 and Table S3.

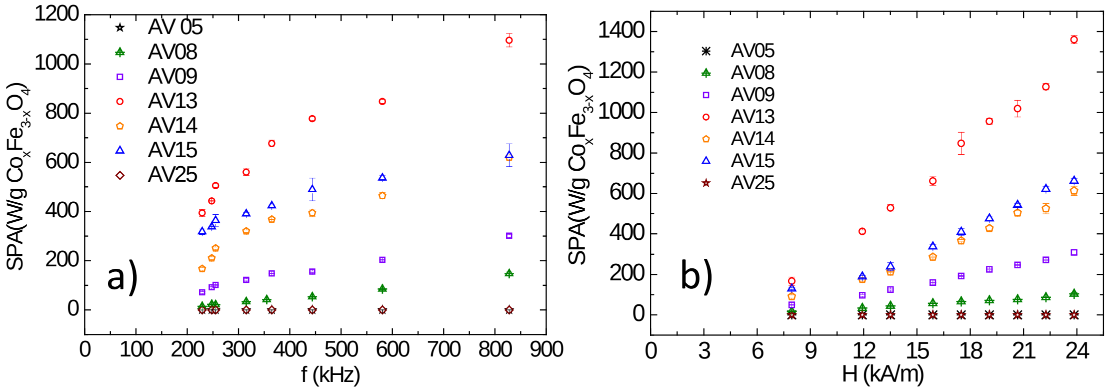

Figure S8: a) Experimental dependence of SPA vs. frequency measured at H_0 =18.5 kA/m. b) Experimental dependence of SPA vs. field amplitude, measured at f = 580 kHz.

Table S2. SPA of samples dispersed in hexane, measured at 18.5 kA/m for different frequencies.

Sample	<d> (nm)	*f* (kHz), H= 18.5 kA/m 229	248	255.5	315	365	444	580	828
AV05	5	-0.11(0.05)	-0.03(0.04)	0.03 (0.04)	0.02 (0.03)	0.1(0.01)	0.1(0.01)	0.18(0.01)	0.02(0.05)
AV08	8.8	15 (1)	23 (2)	22 (2)	33 (3)	42 (1)	53 (2)	85 (3)	147 (7)
AV09	9.5	71 (4)	92 (3)	101 (2)	122 (4)	148 (1)	155 (3)	204 (2)	302 (5)
AV13	13.3	394 (14)	443 (1)	505 (8)	560 (14)	676 (14)	777 (8)	847 (8)	1096 (27)
AV14	14.3	167 (6)	210 (5)	251 (5)	320 (5)	368 (1)	394 (15)	464 (13)	619 (10)
AV15	15.8	318 (11)	338 (1)	364 (23)	390 (6)	424 (7)	490 (46)	536 (12)	629 (46)
AV25	25	0	0	0	0	0	0	0	0

Table S3. SPA of samples dispersed in hexane, measured at 580 kHz for different values of the magnetic field amplitude.

Sample	<d> nm	H(kA/m), *f* =580 kHz 7.9	11.9	13.5	15.9	17.5	19	20.6	22.2	24
AV05	5	0	0	0	0	0	0	0	0	0
AV08	8.8	18 (2)	34 (1)	44 (2)	57 (1)	66 (3)	72 (1)	77 (3)	87 (2)	104 (6)
AV09	9.5	49 (1)	97 (5)	125 (3)	160 (2)	192 (2)	225 (3)	246 (2)	271 (4)	309 (11)
AV13	13.3	166 (20)	412 (8)	529 (16)	661 (21)	847 (55)	956 (13)	1019 (41)	1127 (16)	1360 (21)
AV14	14.3	91 (8)	176 (9)	212 (4)	286 (4)	367 (6)	428 (11)	504 (13)	525 (25)	613 (23)
AV15	15.8	130 (4)	189 (6)	238 (20)	337 (12)	410 (8)	476 (12)	543 (7)	622 (13)	661 (13)
AV25	25	0	0	0	0	0	0	0	0	0

Supplementary Information 5

SPA dependency on the viscosity

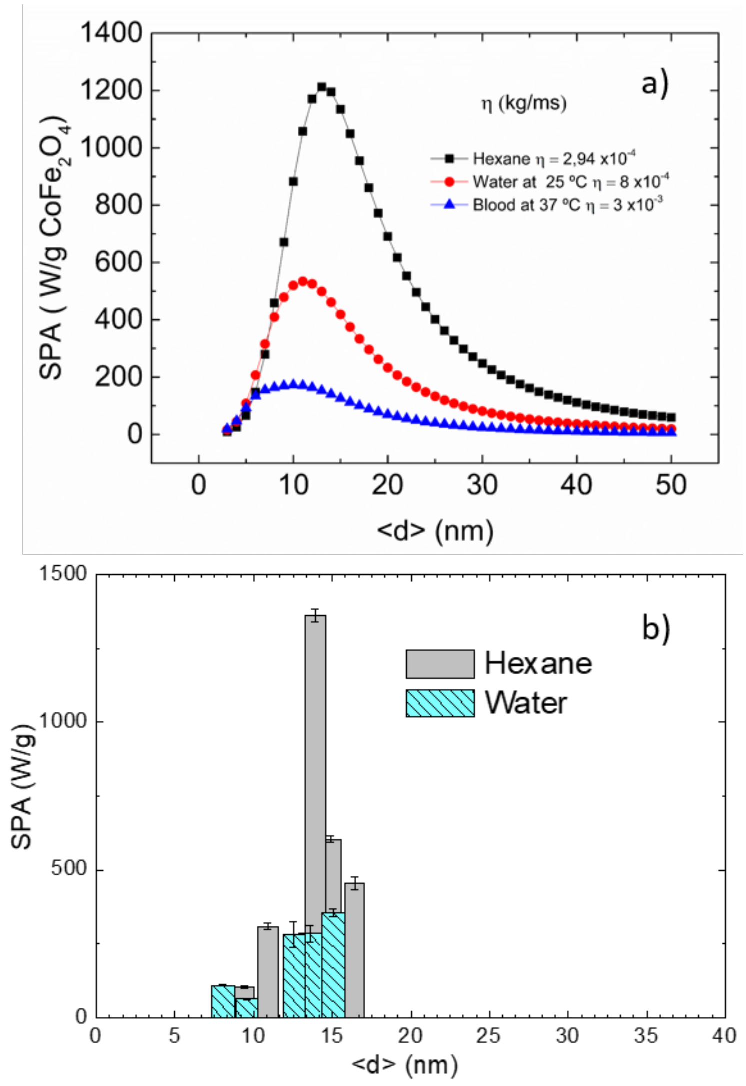

Figure S9 : a) Calculated SPA vs <d> of cobalt ferrite nanoparticles dispersed in hexane, water and biological fluid (blood) respectively (the simulations were done using as input the physicochemical parameters of the sample AV13 derived experimentally) b) Experimental SPA vs. <d> for cobalt ferrites dispersed in water and hexane. The results correspond to samples AV05, AV08, AV09, AV13, AV14 and AV15, applying a magnetic field given by H=24 kA/m and f =580 kHz.

Supplementary Information 6: Cytotoxicity and MNPs cellular uptake

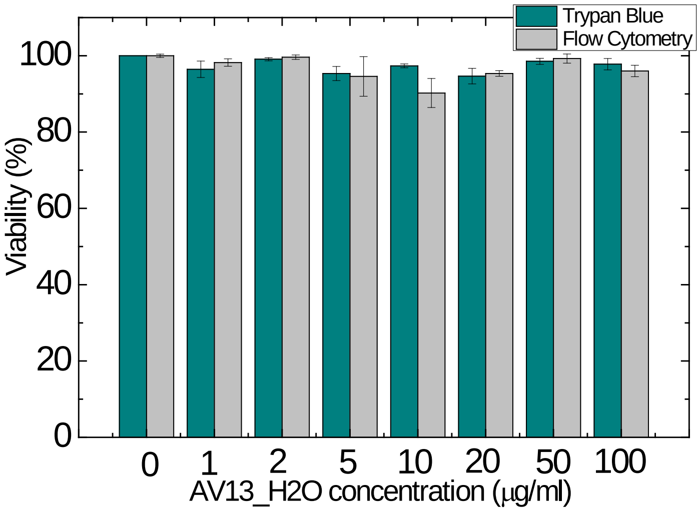

Figure S 10: Viability of SH-SY5Y cells incubated with Co–ferrite MNPs (sample AV13_H2O) for increasing MNP concentrations. Results from Trypan Blue (green bars) and flow cytometry (grey bars) assay.

All the experiments presented in this section have been performed using the sample AV13_H2O. The experiments with MNPs-cell cultures and cell viability were performed by triplicate. Figure S10 shows that for all concentrations the viability remained above 90 %, indicating a very small cytotoxic effect up to the largest concentration used. Once we determined the level of cytotoxicity of our MNPs, we evaluated the amount incorporated into the cells as a function of the MNP concentration added to the cell culture. The mass of MNPs inside the samples was determined by measuring the magnetization of the cell-MNPs culture, using a Vibrating Sample Magnetometer (VSM). As reference for the absolute magnetization value, we used a sample of pure AV13_H2O colloid. Figure S11 shows the measured M(H) isotherms, including also the M (H) data from MNPs-loaded cells with different MNP concentrations (Figure S11 a-b)

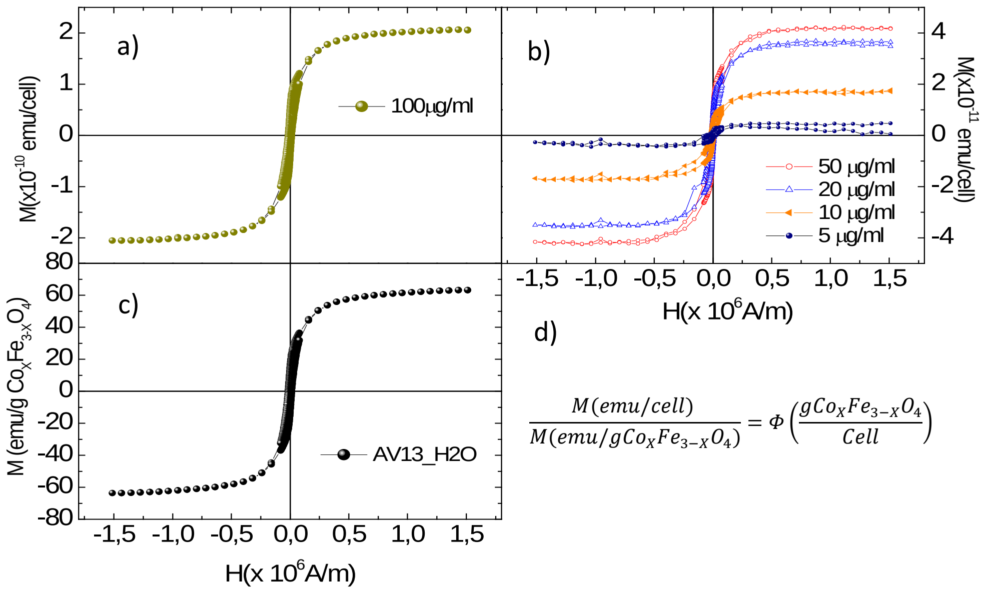

Figure S11: Room temperature M(H) data of cell aliquots loaded with different concentrations of MNPs. a-b) Magnetization corresponding to cells loaded with different concentrations of MNPs (100, 50, 20,10,5 μg/ml respectively) c). M (H) for pure AV13_H2O colloid. d) Expression used to calculate the amount of MNP per cell.

The magnetization signal of the control sample (i.e, cells without MNPs) was subtracted in each case to extract the total amount of MNPs incorporated into the SH-SY5Y cells as a function of the total mass of MNPs added (Figure S12a), using the expression in Figure S11. Figure S12b) shows the amount of MNPs incorporated per cell (each well contained ≈$1x10^6$ cells). The values are within the 0.25-3.5 pg/cell range. For a MNP mass up to 125 μg, a linear increase of the uptake of MNPs was found (see Figure S12a). The data could be fitted with the linear function $y = Bx + A$, with B= (0.02 ± 0.004). This value of B implies that after incubation for 24 h the cells were able to incorporate only 2.0 % of the particles added to the culture media, which is lower than the previously reported values of similar particles.[3] Fortin *et al.* used $CoFe_2O_4$ nanoparticles with carboxylate groups (negative charge) on their surface to incubate with human prostatic adenocarcinoma cells (PC3). [4] They reported MNPs uptake values of 25-30 pg/cell at the intercellular level. The percentage of internalized MNPs could be enhanced by the increase of the incubation time. The value obtained when the incubated cells with 250 μg of MNPs, may be because for such amount of MNPs these are not only inside the cytoplasm but also on the cell membrane. In fact this has been confirmed by the SEM image shown in Figure S13a. This top view image shows the MNP aggregates

on the cell membrane. No aggregates were detected on cells incubated with lower MNP concentrations as for example the ones shown in Figure S13b (MNPs mass = 20 μg).

The low MNPs uptake found in our experiments suggests that in addition to surface charge, the affinity for the MNPs could also depend on the specific cell line involved. Previous studies on Fe_3O_4 MNPs and the same SH-SY5Y cellular line of our experiments, [5] it was reported that the cell uptake was higher (around 60%) for MNPs with positively-charged surface than for negatively-charged MNPs. This difference suggests a specific interaction mechanism between the functional groups on the MNP surface and the cell membrane. The negative charge on the MNP surface induces lower adhesion to the cell membrane, affecting their internalization by the cell. In our experiments, the AV13_H2O MNPs have negatively charged surface groups due to the PMAO coating, and therefore a low affinity of the cell membrane would be the reason for the low uptake observed. Moreover, the uptake might also depend on the protein corona formed onto our MNPs when in contact with the culture medium.

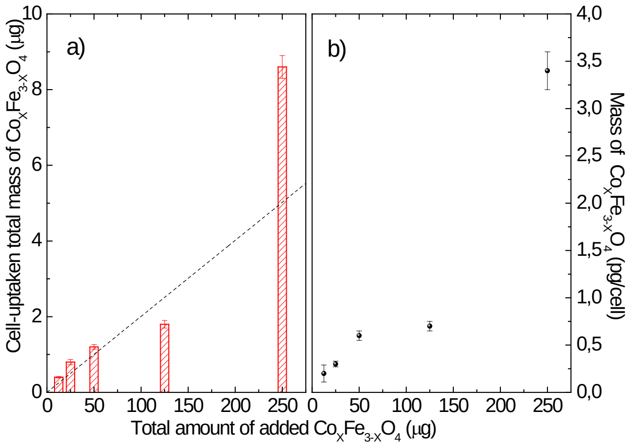

Figure S12 : a) MNP mass incorporated by the total amount of cells vs total mass of added AV13_H2O MNPs and (b) MNPs mass incorporated per cell vs. total mass of added AV13_H2O MNPs

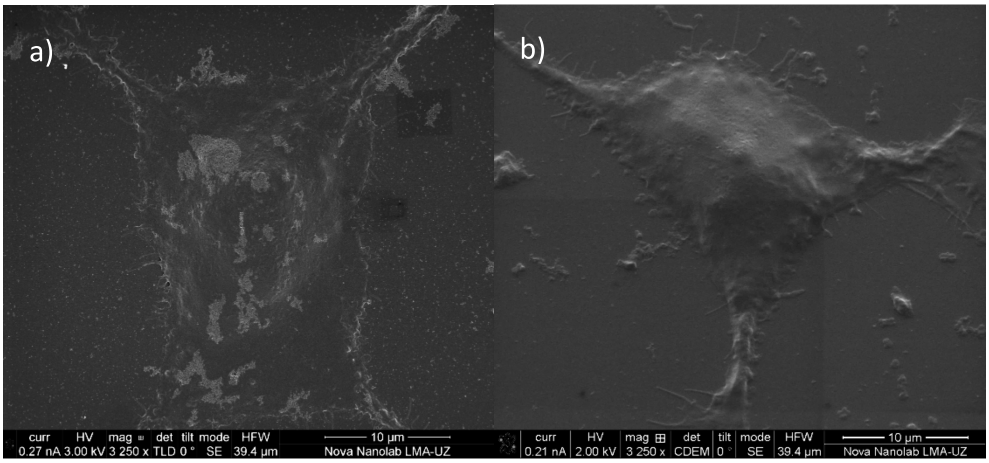

Figure S13: SEM images of SH-SY5Y cells cultured with MNPs in a concentration of (a) 100 µg/ml (b) and 20 µg/ml.

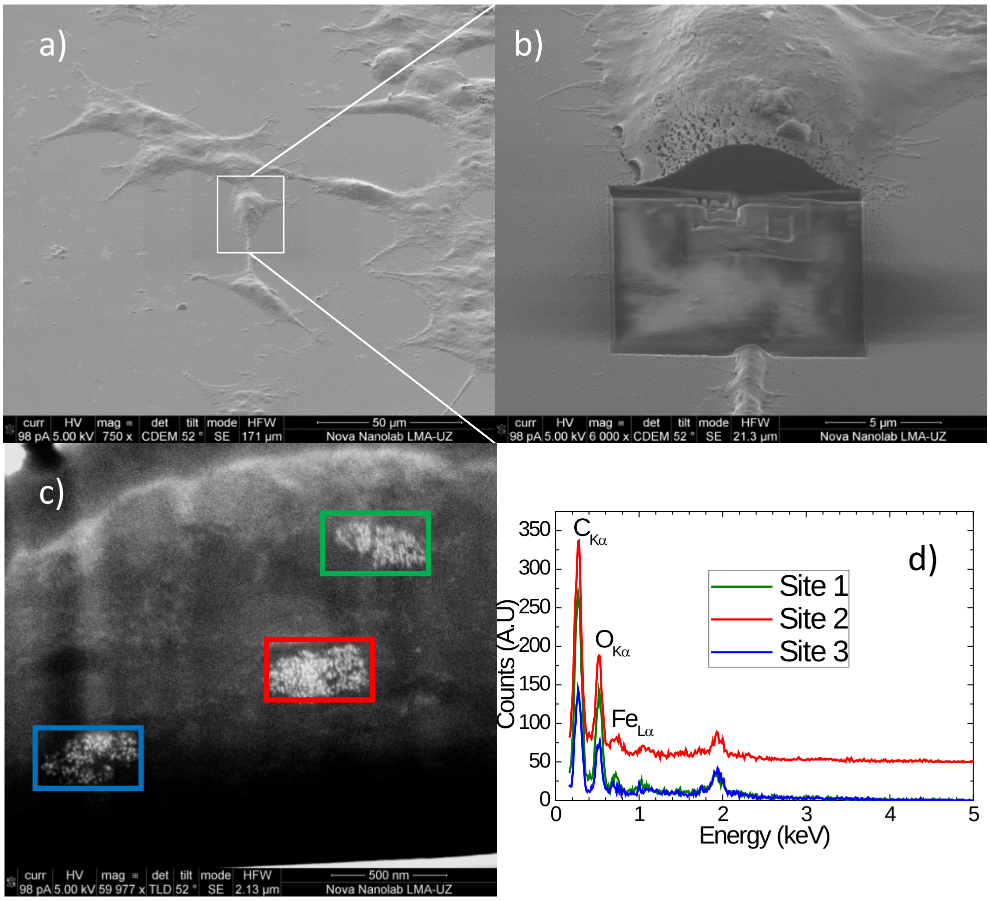

Figure S14: a) SEM/FIB image of SH-SY5Y cells cultured with MNPs in a concentration of 100 µg/ml. b) View of a transversal slice obtained by ion milling. c) Zoom of the transversal slice in BSE mode showed in b), showing MNP aggregates within the cytoplasm. d) EDS elemental analysis confirming the Fe presence in the MNPs core.

Supplementary Information 7: Slice and view process (S&V)

The slice and view process consists in acquiring a sequence of cross sectional images spaced evenly through a region of a bulk specimen and reconstructing those two-dimensional images into a three-dimensional representation of the sampled volume. The samples studied in our case consist of cells containing our MNPs. These cells are embedded in a resin. The sample is fixed on a sample holder with silver paint, and coated with Pt in order to avoid electrical charge on the sample. A cutting plane is opened in the surface of the resin block, at the preselected region of interest (see Figure S15 a). During the S&V process, a protective coating is deposited over the intended milling area, to protect the surface features or to ensure that a potentially mobile feature remains fixed. A mark for shape recognition is made on the corner of the surface (see Figure S15 b)

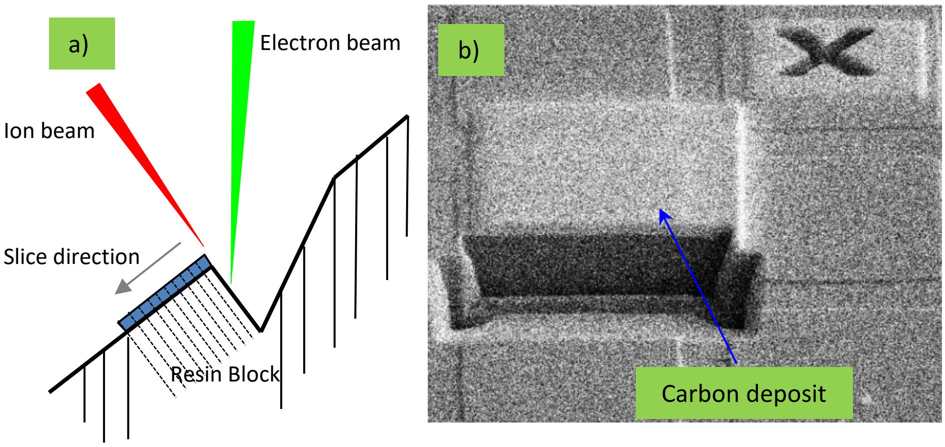

Figure S15 a) Schematic diagram of the the slice and view process by the dual beam. Figure adapted from ref.[6] b) Image of a sample surface showing the C deposit to protect the milling area.

A thin section between 10-30 nm it is milled off by the ion beam and with the electron beam, and a freshly generated plane it is imaged. By repeating this process, a 3D data set is acquired and with these data, the object under investigation is generated. A detailed overview about the automated acquisition of images with FIB-SEM using this process has been reported by Bushby et al[6] and recently an upgraded process was reported by Kizilyaprak et al.[7]

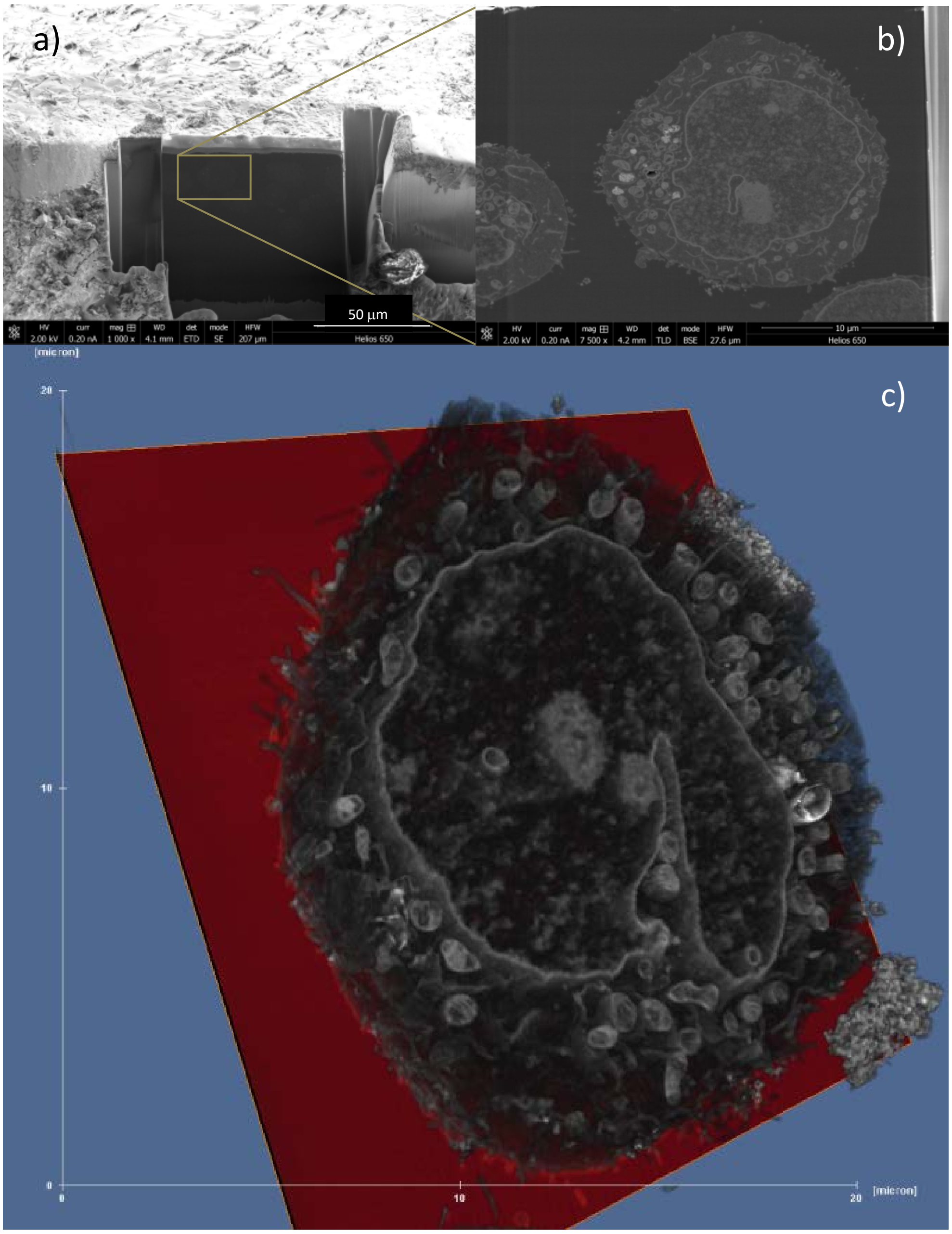

Figure S16: a) Detail of the work zone after FIB milling, b) image of a cell in the region selected in a). c) 3D reconstruction of the cell in b) from images acquired by the S&V process.

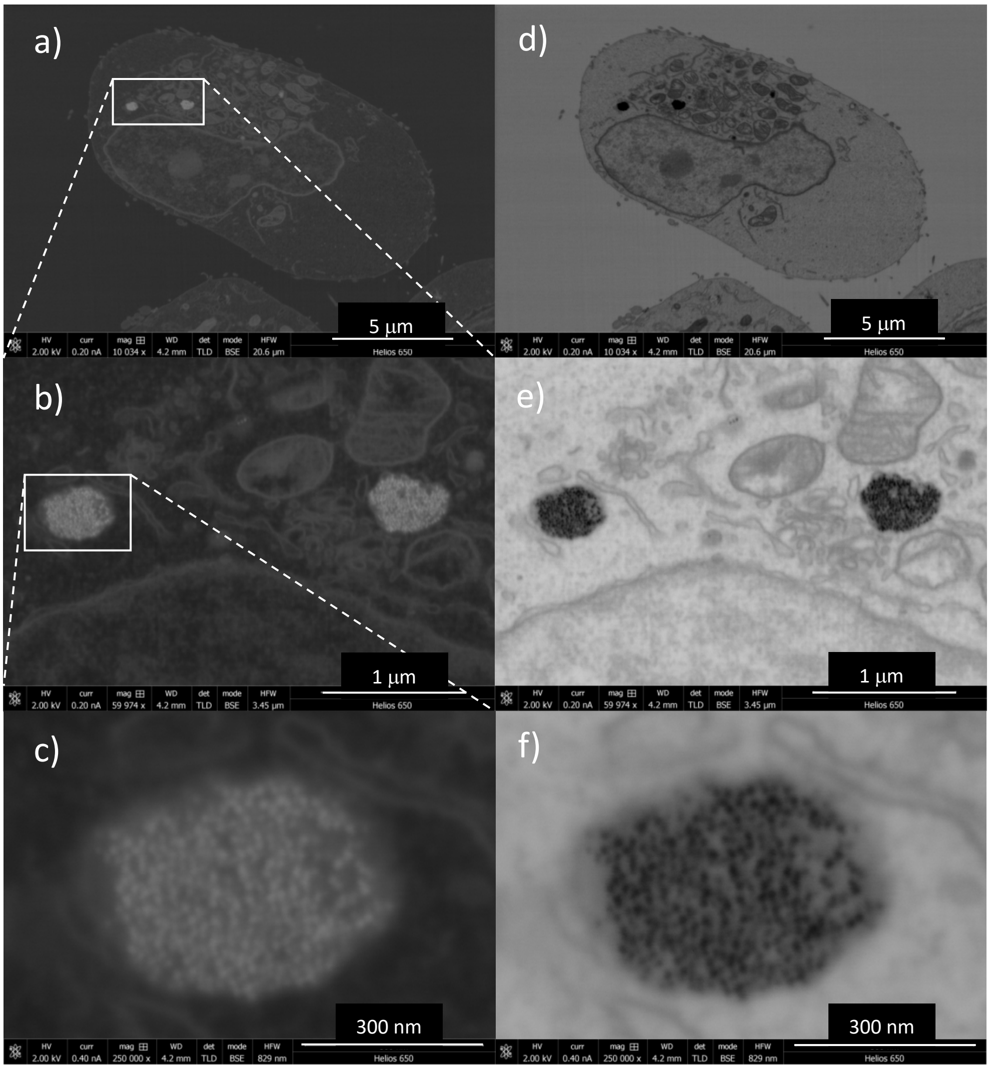

Figure S17: a-c) BSE-FIB-SEM images of cells found in different regions of the milled sample surface. d-f) BSE images with inverted contrast, in which MNP aggregates inside of vesicles are clearly observed.

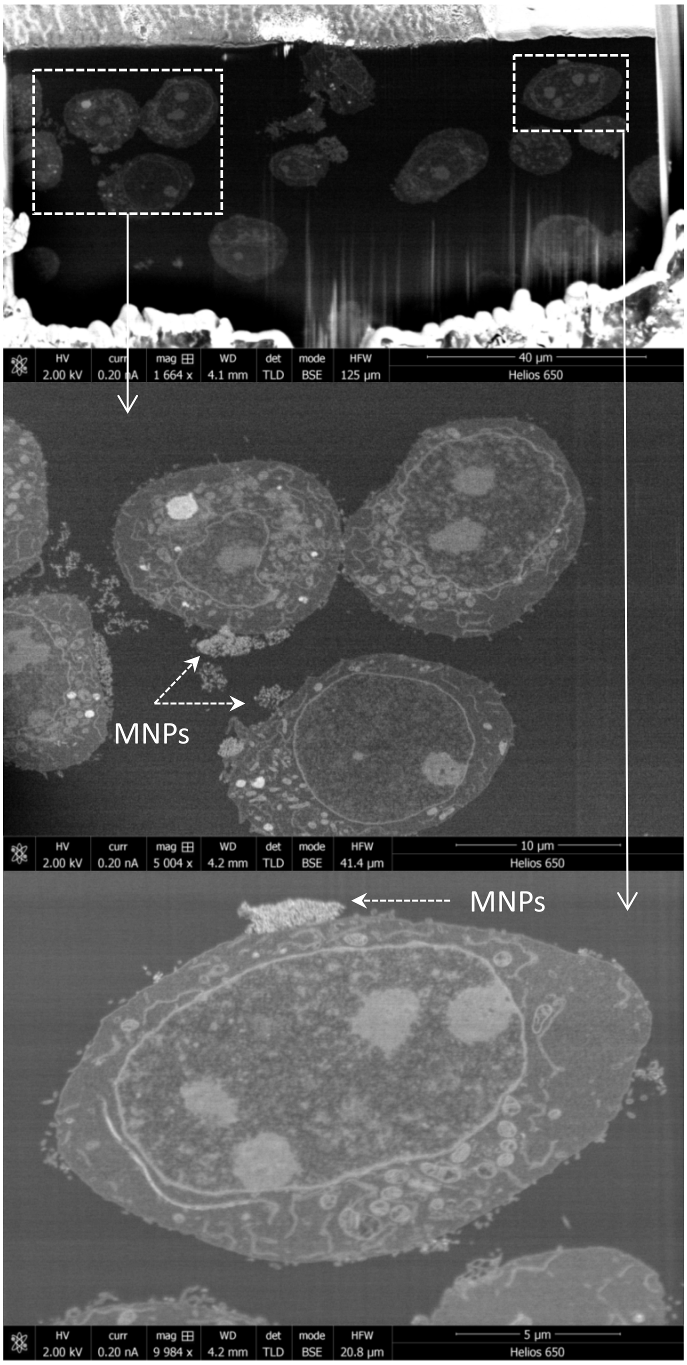

Figure S18: SEM-FIB images of cells incubated with MNPs at 100 µg/ml a) SE-Image of the surface of the resin block after the milling process b-c) BSE-image magnification of the area selected in a) where MNPs are observed in the cell membrane.

REFERENCES

1. T. E. Torres, E. Lima, A. Mayoral, A. Ibarra, C. Marquina, M. R. Ibarra and G. F. Goya, *Journal of Applied Physics*, 2015, **118**.
2. R. E. Rosensweig, *Journal of Magnetism and Magnetic Materials*, 2002, **252**, 370-374.
3. H. M. Joshi, Y. P. Lin, M. Aslam, P. V. Prasad, E. A. Schultz-Sikma, R. Edelman, T. Meade and V. P. Dravid, *Journal of Physical Chemistry C*, 2009, **113**, 17761-17767.
4. J.-P. Fortin, F. Gazeau and C. Wilhelm, *European Biophysics Journal with Biophysics Letters*, 2008, **37**, 223-228.
5. M. P. Calatayud, B. Sanz, V. Raffa, C. Riggio, M. R. Ibarra and G. F. Goya, *Biomaterials*, 2014, **35**, 6389-6399.
6. A. J. Bushby, K. M. Y. P'ng, R. D. Young, C. Pinali, C. Knupp and A. J. Quantock, *Nature Protocols*, 2011, **6**, 845-858.
7. C. Kizilyaprak, A. G. Bittermann, J. Daraspe and B. M. Humbel, *Methods in molecular biology (Clifton, N.J.)*, 2014, **1117**, 541-558.
